# HOW SIGNALING GAMES EXPLAIN MIMICRY AT MANY LEVELS: FROM VIRAL EPIDEMIOLOGY TO HUMAN SOCIOLOGY

**DOI:** 10.21203/rs.3.rs-51959/v1

**Published:** 2020-08-06

**Authors:** WILLIAM CASEY, STEVEN E MASSEY, BUD MISHRA

**Keywords:** Müllerian mimicry, Batesian mimicry, cue mimicry, mimicry ring, signaling game, molecular mimicry, SARS-CoV-2, COVID-19

## Abstract

Mimicry is exhibited in multiple scales, ranging from molecular, to organismal, and then to human society. ‘Batesian’ type mimicry entails a conflict of interest between sender and receiver, reflected in a deceptive mimic signal. ‘Müllerian’ type mimicry occurs when there is perfect common interest between sender and receiver, manifested by an honest co-mimic signal. Using a signaling games approach, simulations show that invasion by Batesian mimics will make Müllerian mimicry unstable, in a coevolutionary chase. We use these results to better understand the deceptive strategies of SARS-CoV-2 and their key role in the COVID-19 pandemic. At the biomolecular level, we explain how cellularization promotes Müllerian molecular mimicry, and discourages Batesian molecular mimicry. A wide range of processes analogous to cellularization are presented; these might represent a manner of reducing oscillatory instabilities. Lastly, we identify examples of mimicry in human society, that might be addressed using a signaling game approach.

## 1. The imitation game: mimicry and signaling game theory

Niccolò Macchiavelli has been much maligned among philosophers for emphasizing the utility of deception, in his book *Il Principe*. However, deception – along with cheap and imitative signaling – turns out to be rather natural when rational agents strategically interact in information asymmetric contexts. In this paper, we formally study the utility of deception, from a signaling games perspective, focusing on mimicry and its universal occurrence, in natural and artificial worlds.

Deception can occur because of information asymmetry, which refers to incomplete information regarding a situation or object and differential states of knowledge held by participants. We suggest that the existence of information asymmetry in nature has deeply affected fundamentals of the genome, organismal biology, and human society and its institutions. Molecular, organismal and cultural evolution are all profoundly influenced by information asymmetry. Here we attempt to unify its effects on all three evolutionary processes, via signaling game theory.

A common form of deception is that of deceptive mimicry. ‘Mimicry’ refers to imitation, and is expected to bring fitness benefits to the mimic. ‘Batesian’ mimicry was the first type of mimicry to be formally described, and involves a ‘mimic’ organism that imitates a ‘model’ organism, in order to deceive a third organism, the ‘dupe’. Typically, a non-toxic species will mimic a toxic species, deceiving a potential predator into wrongly avoiding the non-toxic mimic [1]. Batesian mimicry implies a loss of fitness of the dupe, because it is deprived of a meal, and of the model, given that the value of the warning signal to potential predators is diluted.

One way of reducing Batesian type mimicry and establishing the honesty of a signal is through the use of costly signals. A significant price is involved in the creation of a costly signal, thus deterring mimicry and other forms of deception, as the signal is prohibitively expensive to imitate [2] [3], thus the signal reveals the true type of the sender.

Mimicry may also be cooperative (mutualistic), when two organisms converge on a common signal that is sent to a third organism, to the benefit of all three, this signaling is termed ‘Müllerian’ mimicry [4]. This type of mimicry was originally characterized for toxic animals that share a common warning signal to potential predators. This is easier for a predator to learn, in turn benefiting the animals that send the warning signal. This type of mimicry is non-deceptive, given perfect common interest between the participants.

When more than two organisms share a common signal in a cooperative fashion, they may form a ‘Müllerian’ mimicry ring. There are only a few described examples of mimicry rings, a well-known example is that of bees and wasps (Figure 6a), which share black and yellow markings as a warning of toxicity. Further examples of mimicry rings are identified in this work.

A third type of mimicry is termed ‘cue’ mimicry [5]. A cue is an observable feature that may be inanimate, or of biological origin, and is *non-strategic*, not being intended as a signal. A signal in contrast, has a communication purpose, or meaning [6], and can be regarded as *strategic*. In cue mimicry, a cue is mimicked by an organism, typically to deceive another organism, which could be predator or prey. This can involve blending into the background (crypsis or camouflage). Table 1 gives examples of the different aspects of mimicry observed in biotic systems.

Signaling game theory provides an ideal framework for analyzing the different types of mimicry, providing a better understanding of its evolution and purpose. Signaling games involve an incomplete information setting, that results in the transmittal of a strategically chosen signal between two players, from a sender to a receiver, resulting in an action by the receiver [18]. The strategically chosen action by the receiver results in an increase in utility (analogous to organismal fitness), which is distributed to the players. If the signal is honest, then utility accrues to both the sender and the receiver; here the signal is mutually beneficial. If the signal is deceptive however, then the sender experiences an increase in utility, but the receiver experiences a shortfall in its expected utility [19].

‘Replicator’ dynamics refers to the propensity of a player with a strategy that produces a higher utility to preferentially replicate within a population, and introduces an evolutionary aspect to repeated games [20]. Senders with signaling strategies that result in increased utility, or receivers with an action strategy that produces a higher utility, will be more likely to become fixed in the population. Repeated games facilitate learning by the receiver to recognize the signal; this dynamics can occur in the lifetime of the organism, or over generations.

**Fig. 1:**
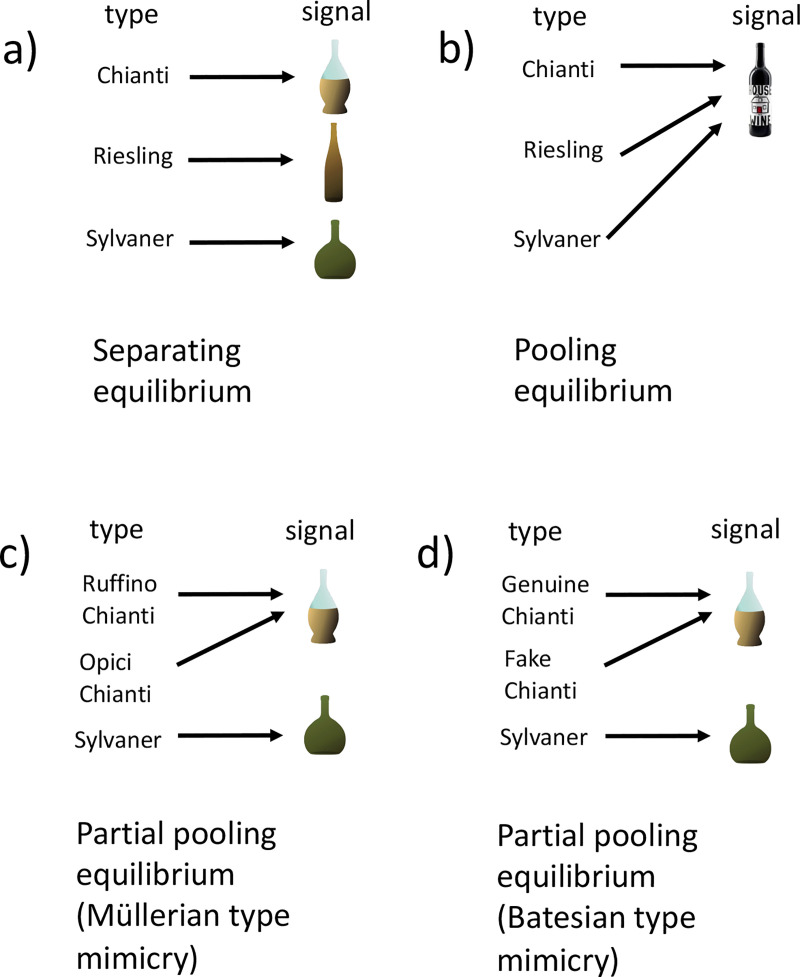
Separating, pooling and partial pooling equilibria. A separating equilibrium (a) occurs where senders of different types send distinct signals, each of which elicits a specific action by the receiver, to the benefit of both sender and receiver [24]. A pooling or babbling equilibrium (b) is where senders of different types send the same signal, and the resulting action of the receiver is always the same. Most relevant to the phenomenon of mimicry is the ‘partial pooling’ equilibrium. Here, two or more senders of different types may send the same signal to a receiver, eliciting the same action, while other senders send different signals. Both Müllerian type (c) and Batesian type (d) mimicry can be represented by a partial pooling equilibrium. The partial pooling equilibrium can represent a common signal that two or more senders have converged upon, to their mutual benefit (Müllerian). Alternatively, it can also describe deceptive signaling by one sender, which imitates or mimics a signal sent by one of the other senders (Batesian). Different types of wine (Chianti, Riesling and Sylvaner) which each possess distinctive wine bottles (the signal) are used to illustrate the different types of equilibria. In (a) the distinct bottles act as a separating equilibrium. In (b) all three types of wine use the same bottle; this is a pooling equilibrium. In (c) different Chianti wineries, Ruffino and Opici, use the same characteristic Chianti bottle; this is cooperative Müllerian type mimicry. In (d) fake Chianti manufacturers use the typical Chianti bottle to deceive customers into buying the wine. This is Batesian type mimicry. Aposematic mimicry represents a special case of partial pooling equilibrium with a pooled signal of toxicity, contrasting with organisms that lack the signal, the action of the receiver being ‘avoid’ and ‘consume’, respectively

Different types of signaling equilibria are associated with different types of mimicry. A so-called ‘separating’ equilibrium (also known as a signaling system/convention, or Lewis signaling game [21]), is where a specific signal from a sender leads to a specific action by the receiver, to mutual benefit. The signal is expected to be honest providing an accurate indication of the type of the sender (Figure 1a). A ‘pooling’ (or ‘babbling’) equilibrium is where the signal has no meaning and does not result in a specific action by the receiver [22] (Figure (1b). Pertinent to mimicry is the partial pooling equilibrium. Here, there are at least three senders, and two of them will send the same signal to the receiver, which results in the same action [23]. Both Müllerian and Batesian mimicry correspond to a partial pooling equilibrium (Figure 1c and 1d, respectively).

**Fig. 2:**
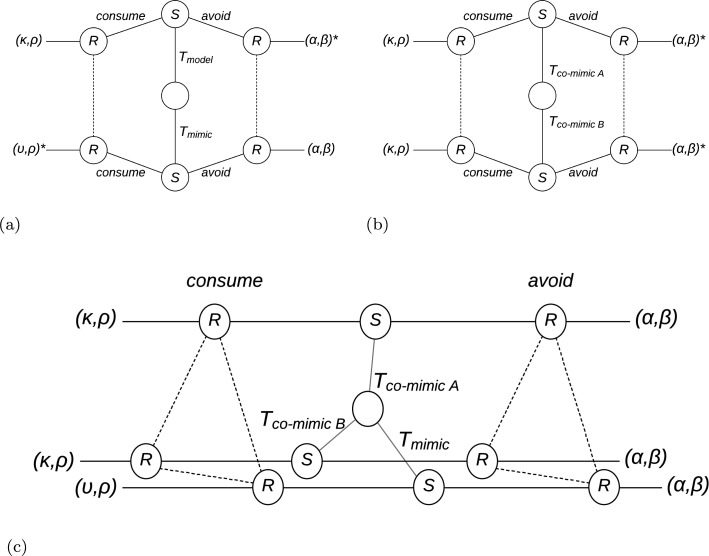
Extensive form decision trees for signaling strategies involving mimicry. Extensive form decision trees show the sequence of interactions between players in a game, and the respective payoffs, depending on the strategies that they follow. Extended form decision trees are shown for a) Batesian type, b) Müllerian type mimicry, and c) Mixed type. The open circle represents a decision taken by nature regarding the type of sender, which may be of two types. Two potential signals may be sent by senders: *avoid* or *otherwise* (consume). The dotted lines indicate that the receiver has incomplete information regarding the identity of the sender, which may be of two (or more) potential types. The receiver has two potential options, *avoid*, or *consume*. The utility payoffs are in brackets (*S, R*), and the Nash equilibrium is indicated by an asterisk. In (a) there are two types of sender, Τ_model_ and Τ_mimic_, corresponding to the model and Batesian mimic respectively. In (b) the sender may be of type Τ_co-mimicA_ or Τ_co-mimicB_, corresponding to two Müllerian co-mimics, A and B. In (c) we show how scenarios are composed to form a mixed type.

Müllerian mimicry rings may be viewed as assuming a star network structure. A Shapley value is generated amongst all the senders and the receiver that comprise the mimicry ring (the Shapley value is a measure of the distribution of utilities amongst players in a cooperative game [25]). An important observation is that Batesian mimicry is *frequency dependent*: the higher the frequency of Batesian mimics in a population, the lesser their fitness advantage. This is because too many Batesian mimics will dilute the value of the signal [26], to the extent that selection processes view better discrimination mechanisms as advantageous over the continued use of the diluted signaling system [27]. Extensive form decision trees for Batesian, and Müllerian mimicry, are shown in Figure 2.

## 2. Methods

We construct a mathematical model to study signal evolution in these informational asymmetric scenarios. We start by formalizing population structure as an ensemble of communicating types (species), with each type representing a population of agents. Next we define the space of signaling strategies, the signaling game encounters, and the differing rewards for encounters due to type and receiver action. We will assume a rudimentary decision function for the uninformed receiver that imposes a decision surface in signal space. Differing signaling strategies will yield differing rewards from encounters, to guide evolution we assume mechanisms that relate rewards to replication rates within type. Strategies that gain higher reward will spread at a quicker rate than their lower yielding peers. Additionally we incorporate a stationary mutation process that generates new strategies periodically. We thus incorporate natural selection, replication and mutation for each types within an evolutionary game.

With the evolutionary game defined we set the stage for analyzing mimicry forms and their dynamic consequences.

### Population Structure and Predation Rewards

Agents are organized into type groups. Aside from constraining replication, types will determine outcomes of certain receiver actions during encounters. For example, as predators encounter other organisms in the wild, predation decisions are made using markings (signal). Poor metabolic outcomes are possible and the organisms’ type will be the most important factor if predation occurs. The simplest interesting scenario is when two types have related markings but one is toxic while the other is nourishing.

We denote the population of agents who are type *x* as *τ*_*x*_, the population size of type *X* will be denoted *N*_*x*_ = |*τ*_*x*_|. The population structure will refer to the types (and population sizes) within a model. For example if there are *m* types, all agents can be partitioned as $\cup_{j=1}^{m}\tau_{j}$.

The predator’s metabolic reward function for predation outcomes, will depend on an agent’s type. Assuming a predator consumes agent *a*, the reward will be:

$$R(a)=\{\begin{array}{l} r_{1}\;\text{if}\;a\in \tau_{1}\\ r_{2}\;\text{if}\;a\in \tau_{2}\\ \cdots \\ r_{m}\;\text{if}\;a\in \tau_{m} \end{array}$$

In our simplest scenario, *m* = 2, the first type toxic and the second type nourishing is represented by values: *r*_1_ < 1 < *r*_2_. The metabolic reward of 1 can be thought of as neither loss nor gain.

### Signaling Strategy and Predation Decisions

The signaling space is a set of distinguishable features, within the receiver’s sensory and sender’s combined repertoire of expression. All relevant signals can be represented within a *signal space*, a vector space $\Gamma ={\mathbb{R}}^{N}$ for sufficiently large *N*. The signal space may consist of morphological, phenotype, markings, sounds, smells, coloration, or other attributes sensed or communicated that factor into the receiver’s predation decision. For agent *a*, the signaling strategy will be denoted *s*(*a*) ∈ Γ.

Additionally, we will use the same space Γ (along with a scalar threshold) as a parameter space for the receiver’s decision function. In our simple scenario we model the predator’s choice of action as: **predation** or **avoidance** by partitioning Γ into various regions which prescribe those actions.

The simplest decision model^1^ for the agent receiver will utilize a reference point *q* ∈ Γ termed the *avoidance feature*, this will identify the center of an *avoidance region* in Γ. Using the cosine distance, $\mathrm{cosd}(x,y)=1-\frac{\langle x,y\rangle }{\left\|x\|\|y\right\|}$, the avoidance region is symmetric around the avoidance feature, with radius determined by the threshold parameter *δ* ∈ [0, 2]. This results in the decisions function:

$$A(s|q,\delta )=\{\begin{array}{l} \mathrm{avoid},\;\text{if}\;(\text{cosd}(s,q)<\delta );\\ \mathrm{predation},\;\text{otherwise.} \end{array}$$

When encountering an organism, any signal received within the avoidance region will be instinctively avoided, otherwise predation instincts prevail.

### Signaling Game and Metabolic Objectives

Signaling games, start with nature, that selects the sender type. Nature provides agent encounters, assigning the type (identity) for the organism (sender) and receiver (predator). Next the sender transmits a message *s* ∈ Γ. The receiver having received the sender’s message but without certain knowledge of sender’s type, must select an action. Differing rewards occur depending on the combination of nature’s selection of types, sender’s signal, and receiver’s action.

Abstractly this payoff function is represented by:

$$\underset{sender}{\underset{︸}{T\times \Gamma }}\times \underset{receiver}{\underset{︸}{A\times T}}\to \underset{rewards}{\underset{︸}{\mathbb{R}\times \mathbb{R}}},$$

where the first two symbols represent the sender’s type and signal, the following two symbols are the receiver’s action and type (also selected by nature if there are multiple receiver types), finally the right-hand side represents the rewards for sender and receiver, in that order.

The signaling game in our scenario can be viewed most clearly during an encounter scenario: When predator encounters an organism that sends a certain signal (markings or coloration). The predator receiver, uncertain of the sender’s type, selects an action, predation or avoidance, resulting in dramatic and differing health outcomes.

The repertoire of types (formalized as population structure), that nature presents to predators during encounters, will play a significant role in signaling game outcomes. We illustrate how this affects the signaling game structure in figure 2 where we illustrate extensive forms for signaling games with a variety of population structures. We provide a general reward matrix (in table 2) for game outcomes, this table includes the types used within the population structures studied. We use these types to create various ensembles for analysis, and consider the types of mimicry they express. Our results suggest that the population structure and predation reward function are critical to understanding how mimicry is formed, maintained, and destroyed in populations.

To keep our descriptions as simple as possible we will consider only one type of predator in the study, the outcome from signaling games is given by the reward function U:T×Γ×A, specifically:

$$U(t,s,A(s|q,\delta ))=\{\begin{array}{l} (1,1-\eta ),\;\text{if}\;\mathrm{cosd}(s,q)<\delta \;\mathrm{avoid};\\ (\epsilon ,R(t)),\;\text{otherwise.} \end{array}$$

The equation above may be further modified by the parameter such that 0 < *ϵ* < 1 representing the reduction in fitness (reproductive likelihood) for a sender organism consumed during the encounter. Values of R(t) apply for predation; the small deduction *η* < 1, accounts for metabolic loss and deferred replenishment for avoidance.

### Best receiver action

Within a population of replicating strategies guided by evolution, more successful strategies will have increased replication rates. To explore these dynamics further we consider how the utility optimization implicitly depends on the statistical distribution of types. We consider the distribution *μ* which measures the probability of type given a specific signal *s*.

Letting:

$$\Theta (A|s)=\sum_{t\in T}U_{2}(t,s,A(s|q,\delta ))\mu (t|s),$$

with *U*_2_, the receiver’s utility, and *μ*(*t*|*s*), probability of type *t* given signal *s*. The best response may be taken as an argument policy maximization which seeks the best action as yielding the highest reward averaged over all agents of all types:

$$\underset{A}{\mathrm{Argmax}}\;\Theta (A|s).$$

By integrating over the signal space, calculation of the best response is revealed as a geometric problem:

$$\underset{q,\delta }{\mathrm{Argmax}}\;\sum_{t\in T}R(t)\left(1-\lambda_{\left(q,\delta ,t\right)}\right)+(1-\eta )\lambda_{\left(q,\delta ,t\right)},$$

where *λ*_(*q,δ,t*)_ is the proportion of *t*-type population with signaling strategy (signal or markings) contained in the avoidance region (*q, δ*). Notice the interesting case for mixed types, for example if *r*_1_ < 1 − *η* < 1 < *r*_2_ in our simplest scenario. Selection of parameters for best response is a nontrivial instance of the geometric knapsack problem. The dynamic evolutionary games further places dependence on frequency or distribution of signals. The geometric interpretation gives a sense of the type of distributed co-optimization as it occurs during evolutionary games, and provides an analogous geometric problem involving reinforcement and adversarial learning.

### Evolutionary Games

**Fig. 3:**
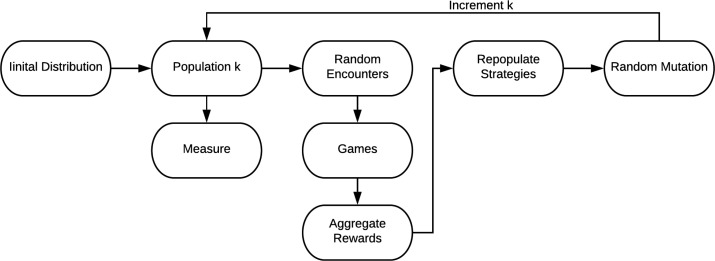
The evolutionary game defines evolving objectives for signaling strategies. The process measures signaling strategies by their metabolic/protection rewards. The process guides evolution by constructing a relation between strategic rewards and reproduction implemented with statistical boosting. The process architecture is simple, but worth noting that each type in the population structure forms a component in the evolution processes. During the encounter phase the type components intermingle with signaling games. Accordingly the strategic objectives are dynamic, dependent on frequency (of strategies used by other types) and can behave in complex ways.

In evolutionary games the dynamic evolution of strategies within a population is considered. We illustrate the phases of the evolutionary game in figure 3 and summarize the three main steps as: *meet*, *mate (& repopulate)*, and *mutate*. Each type in the population structure is a component in the evolution processes, the repopulate and mutation phases will be self contained to the type component. The encounter phase intermingles the agent types coupling the component processes with signaling games that define the evolving objectives and problem solving requirements linking outcomes to replication rates.

#### Initialization

We will define a constant size population of agents for each type *t* as *n*_*t*_. We use an initial distribution to generate *n*_*t*_ pure strategies and assign those to the agents of each type. We set the loop count *k* to be zero. *Measure*: for each time step *k* we record measures of the population to study empirically the evolving distribution of quantities. *Meet/Encounter*: are generated from an *encounter distribution* which generates random pairs with predator and prey agents. *Games* for every encounter, we use the sender type, sender signal, and receiver (predator) avoidance region to calculate the rewards gained from the signaling game. *Score*: for each type, for each strategy i, $\phi_{i}=\frac{g_{i}}{\sum_{j}g_{j}}$ where *g*_*j*_ is the total reward received by all agents who use strategy *j* during encounters. The vector *ϕ* forms a probability distribution over the set of strategies. *Mate/Repopulate*: with details given in the Supplementary Materials, *ϕ* is slightly modified to derive Φ used to re-select *n*_*t*_ strategies (from MULTINOMIAL(*n*_*t*_, Φ)). These new strategies are assigned to the agents of type *t*. Note that this technique is nothing but a simple form of statistical boosting, the better the score the more likely to re-select. Said differently, this phase will prefer to replenish better performing strategies over poorly performing strategies. *Mutation* Next a random mutation process is applied to agents whose strategies are mutated in place. Mutation acts to generate and probe novel strategies which the population can try. *Increment* Time step *k* is incremented and a next population is defined, processing continues from the Measure step.

Under natural selection a mimicry ring appears to be in a stable equilibrium because although mutation allows out of equilibrium signaling, it presumably offers only less benefit and accordingly will not survive for long. Once established in a ring, we expect mimicry to endure and evolve. While a Müllerian ring appears stable in isolation, considerable advantages await Batesian mimics to invade the ring. Under natural selection, such invasions lead to loss of rewards by receivers. As such the question resurfaces as to how many Batesians will it take to destabilize the ring.

The methods outlined are analyzed rigorously to evaluate the subjects of ring formation and robustness (to Batesian invasions) with simulation studies of evolving populations of agents under a variety of population structures.

## 3. Results and Discussion

The evolutionary game, discussed earlier, expresses a range of mimicries. Here we will further illustrate and discuss the surprisingly diverse dynamics expressed for a variety of population structures. We will draw attention to three prominent behaviors: 1) the emergence of Müllerian rings and their stability, 2) the adversarial behavior introduced by Batesian mimics, and 3) the behavior of mixed mode mimicry. We will illustrate how the mixed mode expresses both the emergence of rings and the antagonistic dynamics of Batesian types, but further we study how the Batesian types interact with the ring and how they destabilize it and/or eventually, cause its complete collapse. To investigate this phenomenon we illustrate how the mixed mode system evolves by transitions from one game equilibria to another, and the conditions that trigger transitions within the model. We also draw connections to existing biological studies where our results bear close resemblance to natural phenomena.

### Emergence of Rings

In systems with potential common interest for coordinated behaviors, we observe the emergence of Müllerian rings. Once established we expect the ring to endure and evolve. This dynamic is observed most clearly in systems comprised of only toxic types and predators, such as system (1, 0) and (2, 0) which have respectively one and two toxic types, one predator but zero non-toxic types. In these systems we observe a *signal locking phenomena*; where, the predator and toxic types adapt and hold a purposeful signaling convention. Initialized in ‘babbling,’ where encounters between predator and toxic types invariably lead to predation, either a mutant predator (with advantageous avoidance behavior) or mutant toxic organism (that predator instinctively avoids) will eventually occur. Once such an event occurs, both populations quickly replicate those strategies catalyzed by the increased rewards they offer, as can be seen in figure S5 (b) and figure S9 (b). Figure S6 illustrates the transition and the rapid crossover the populations take. This transition will be driven by the new strategies being favored and boosted in replication. Additionally this will coincide with the abandonment and extinction of many inferior strategies for the singular new one, accordingly, as the population becomes more clonal, we observed a decreased variance in strategies. The temporal requirements for such a transition can be understood as the expected search time for ‘paths to cross.’ Within a few generations a ‘separating equilibrium,’ where the signal is used to distinguish type (e.g., the receiver’s basic problem of determining what to eat) takes over and a Müllerian ring is formed. Once established, we expect the ring to endure, its stability achieved by selective advantage, stumbled upon by mutants probing alternative strategies. Should a mutant strategy break the signaling convention their replication rates are attenuated by the less satisfying outcome. The stability of the ring can be observed in figure S5 and S9; these plots show the cosine distance between the strategies of encountering organisms. Any encounter within the avoidance region will result in the predator avoiding the organism, while predating otherwise. Clearly, the avoidance region is an attractor and absorbing state. Additionally, the avoidance decision can be interpreted to delineate foreground from insignificant or abiotic background to address cue mimicry forms. We emphasize that signal locking need not imply a constant or frozen signal convention (indicated by the motion of centroids in figure S7 and S10). Still this possibility points to an interesting mathematical question; namely, of asymptotic behavior as the ring grows in size.

As the ring endures, substantial advantages await Batesian mimics to invade. Since these invasions weaken the utility of the signaling convention, it raises a critical question: namely, how many Batesians does it take to destabilize the ring entirely.

### Adversarial chase

Systems with conflicting reward structure will exhibit adversarial dynamics, as is observed most clearly in system comprised of non-toxic types and predators, such as system (0, 1) and (0, 2) which have one and two non toxic types respectively, and one predator type but zero toxic types. In these systems we observe an antagonistic chase: the non-toxic types attempt signal locking, however the predator repels any such signal convention by rapidly mutating and moving its avoidance region in response (see figure S8 and S13).

Initialized in ‘babbling,’ the non-toxic type seeks to mutate and rapidly adapt a strategy that predators instinctively avoid. Predators repel selective forces in this direction by attenuating replication of easily duped avoidance strategies. Should a non-toxic mutant dupe all predators into avoidance, predator mutation will eventually ensure a return to predation. Evolutionary events, which could offer a common signaling convention, are no longer sought by all types (as they are when a Müllerian ring forms), so they are no longer the flash-points for rapid adaptation in both the predator and prey class. Rather, in these cases what is good for one class is bad for the other; thus, setting the stage for the antagonistic chase.

Our simulations, as presented here, give the appearance that the predator has greater control, repelling the separating equilibrium (where Batesian mimics would thrive) in favor of the Babbling equilibrium (where predator minimizes loss); however, this notion of which type has greater control will depend on key model parameters. Note that the decision function has an important geometric aspect, with *τ* = 0.2 the avoidance region has far less volume than the predation region, when *τ* = 1.8 we observe that the non-toxic type controls the equilibrium by repelling babbling while maintaining the separating equilibrium.

### Mixed mode behaviors

**Fig. 4:**
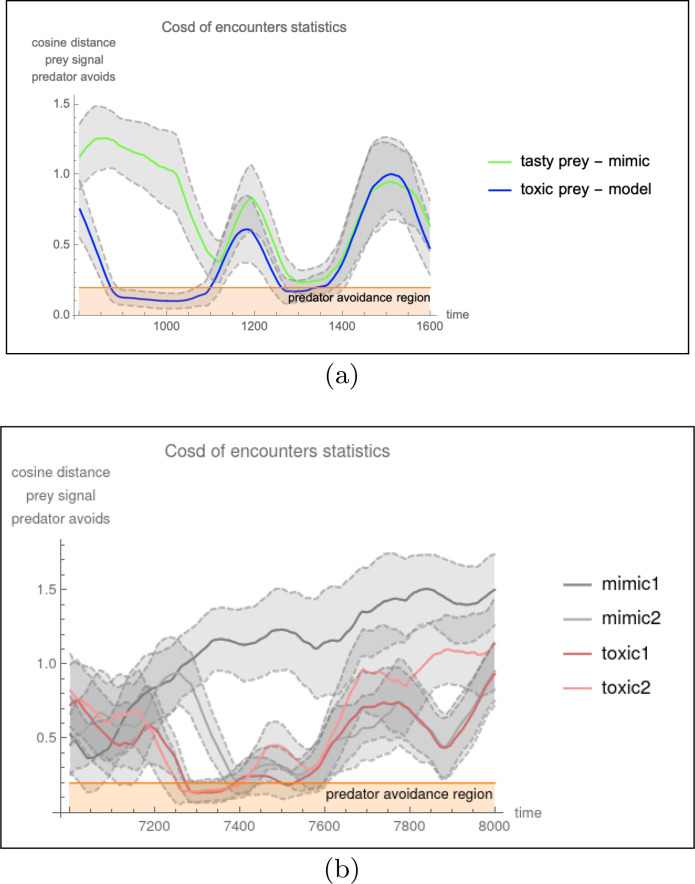
Mixed Mimicry modes are shown to oscillate: A ring is established frequently but invaded by the non-toxic type which destabilizes the ring and precipitates the abandonment of the partial pooling in favor of babbling. When the ring is invaded the partial pooling equilibrium transitioned to a pooling equilibrium rendering the receiver’s discerning strategy into one that is too timid. In (a) a population of one toxic, one non-toxic, and one predatory type evolve signaling strategies over time. In (b) a more complex scenario with a population of two toxic types, two non-toxic types and one predator. For every generation, a set of encounters results in a cosine distance measure between the predator’s avoidance feature and the signaling organism. Plotted on the vertical axis is the average (and variation band) of cosine distance measures between predator and toxic type (blue) and predator and nourishing type (green). The horizontal shaded region (orange) represents the avoidance region, where encounters will lead to avoidance rather than predation. Since avoidance is mutually beneficial to toxic type and predator we observe epochs (measured in hundreds of generations) where the partial pooling equilibrium is stable and separates toxic from non-toxic types. The stability of the signaling system appears to be disrupted and destabilized when non-toxic types signal within the avoidance region.

**Fig. 5:**
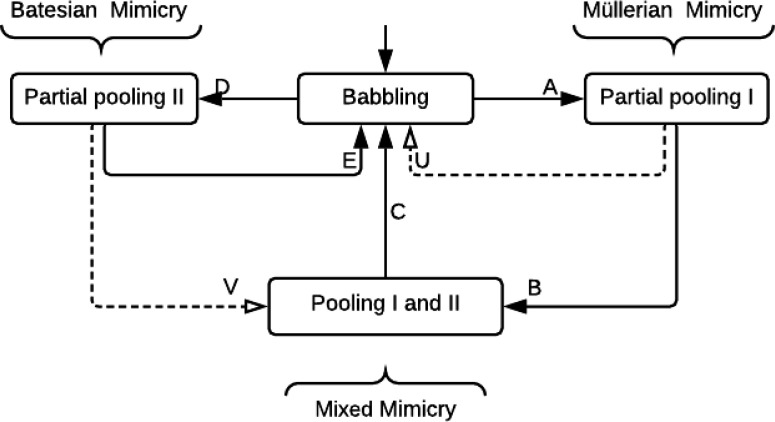
Oscillation in Mimicry modes as described by transitions in evolutionary game equilibria: Using the game outlined in Figure 2 with predator, toxic type, non-toxic type initialized in a babbling equilibrium. Each type leverages mutant and diverse strategies to search signal space. When toxic type and predator are first to lock signals, *transition A* is compelled by the utility seeking behavior of both causing a transition to *partial pooling I*, this is where predator and toxic type establish a signaling convention that is mutually beneficial and increases their utility. We observe that from *partial pooling I*, organisms from non-toxic type will eventually invade leading to *transition B* that yields a higher utility for mimic at the expense of the receiver (predator). This transition leads to a mixed mode where cooperative and Batesian mimicry strategies are simultaneously expressed and the signaling system is in pooling equilibrium. Note that predator has lost average utility from its prior state in *partial pooling I* and could exploit diverse or mutant strategies to return to a babbling state (*transition C*) if the first of such mutants stands to gain utility, as would clearly be the case when the benefits of consuming non-toxic type outweigh the risk of consuming toxic type. It is also possible that from the babbling state the non-toxic type first coalesce to predator’s avoidance feature as identified by *transition D* leading to *partial pooling II*. This outcome is purely deceptive Batesian and will lead to gains for nourishing type and a loss for predator. Since predator can leverage diverse or mutant strategies which forget the avoidance feature *transition E* is clearly possible and preferred as a unitary move by the predator species.

In mixed mode systems with both mutualistic and conflicting reward structures (systems (*n, m*) are ensembles with *n* toxic, *m* non-toxic and one predator type) a novel and important behavior arises. Epochs of familiar behaviors are observed, such as ring formation via signal locking between predator and toxic types (as before in (*n,* 0) systems), as well as adversarial chase between non-toxic type and predator (as before in (0*, n*) systems). But critically, we observe a new dynamic behavior: namely, the destabilizing effects of Batesian (non-toxic) invasions on previously formed rings. Since rings emerge from babbling (as in the (*n,* 0) component), and Batesian invasion collapses rings back to babbling, the system cycles and we observe an oscillator (figure 4) whose main cycle is succinctly understood as transitional, from one game equilibrium to another: ordered as babbling, partial pooling, pooling and back to babbling. We illustrate the equilibria transition graph (in figure 5) for the simplest case (i.e., (1, 1) having one toxic, one non-toxic and one predator type^2^) exhibiting the novel cycle. We generalize the discussion to other more complex scenarios where mimicry rings form.

Initialized to babbling, toxic and predator types (attracted by common interest) seek signal locking and the formation of a ring (Müllerian mode mimicry), a non-toxic type seeks to exploit any avoidance cues which predator instinctively employs (Batesian mode). Once a ring forms, enlistment of additional toxic types strengthen the ring by reinforcing the predator’s reward for its avoidance behavior. As non-toxic (Batesian invaders) types invade the ring it reduces the predator’s reward. The predator’s utility is thus the critical ballast for ring’s stability, and while it may tolerate a certain number of Batesian invaders, there may be a threshold at which the babbling equilibrium is preferred. The transition occurs when a mutant predator modifies avoidance and breaks out of the existing partial pooling equilibrium (or forgetting the inherited avoidance habit) thus putting non-toxic mimics back in play for predation. Because this approach increases metabolic rewards above that of the fully timid strategy held by the majority, the mutant strategy will quickly replicate among the predators.

Our result with mixed mode mimicry is consistent with other available evidence: namely, that models will diverge from mimics in a process of antagonistic co-evolution [28]. Theoretical considerations have indicated a ‘coevolutionary chase’ with a continual process of model divergence and mimic catch-up [29] [30]. Studies that monitor changes over time are scarce, and difficult experimentally – given the time scales necessary to observe multiple cycles of divergence and catch-up. More readily available examples might be found in human society, for example currency counterfeiting has to be countered with periodic introduction of new markings into banknotes. Our simulation study is simple and restricted to understanding the basic dynamics of one ring (anchored by a predator with limited avoidance parameters), however it connects in informative ways to studies that employ frequency dependence or consider multiple rings. For example, frequency dependent selection means that the effectiveness of the mimicry signal is reduced at high frequency, which could break the oscillation. While simulations have shown that invasion by Batesian mimics promote convergence between rings due to the promotion of signal divergence, which means that one ring might become similar to a second ring [31], generally their effects have been little studied. Still the criticality of predator’s reward for ring stability indicates that within a larger networks the type of mean field game which arises from the games described here. In the larger networks organisms join as many rings as possible for protection, while predators dealing with rings cluttered with various levels of deception, make critical ring breaking decisions.

### 3.1. Molecular mimicry, the origin of life and cellularization

Molecular mimicry can be more fully understood within a signaling games framework [14]. The gene for an RNA or a protein macro-molecule can be considered as the sender, while the signal consists of the three dimensional conformation of the expressed gene product. The receiver is the macro-molecule, which specifically interacts with the signal macro-molecule, typically a protein, but could also be an RNA or DNA molecule. An action results from the binding of the receiver macro-molecule with the signal macromolecule, which results in an increase in utility (fitness) for both sender and receiver, if there is perfect common interest between the two. The action might be an enzymatic reaction or conformational change by the receiver macro-molecule. The binding (substrate) specificity of the receiver macro-molecule is analogous to organismal receiver discrimination [32].

This model of a bio-molecular signaling game implies that the first signaling games were played between bio-molecules in the earliest life forms, once the bio-molecules were large enough to exert specificity [33] [14] [34]. This in turn implies that *‘meaning’* first arose from the primordial soup, as the first signal was strategically sent, between a pair of replicating macro-molecules. However, immediately ‘meaning’ first arose, it then became susceptible to deception, effectively the original sin.

mRNAs and tRNAs, may be regarded as Müllerian co-mimics, given that they typically have perfect common interest with each other. All mRNA 5’ leaders and 3’ polyA tails may be regarded as signal co-mimics of each other, the receiver being the translation initiation apparatus. In this sense, the protein coding portion of the genome may be regarded as an instantiation of Müllerian mimicry.

**Fig. 6:**
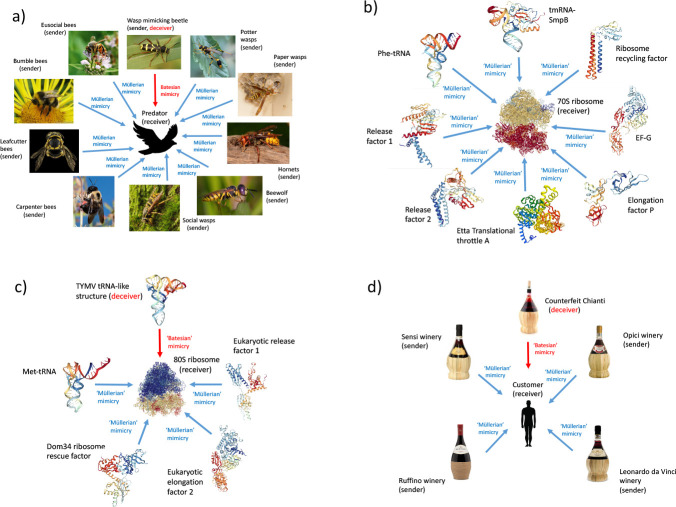
Müllerian mimicry rings at multiple levels. The figure shows a) an organismal Müllerian mimicry ring (adapted from [14]); b) a bacterial Müllerian-type tRNA mimicry ring; c) a eukaryotic Müllerian-type tRNA mimicry ring; d) an economics Müllerian-type mimicry ring formed by Chianti wines. (a) shows a wide variety of bees and wasp species. These all possess a venomous sting, and share a common warning signal, black and yellow markings, which defines them as Müllerian co-mimics. The *Clytus arietis* beetle is a Batesian mimic, as it shares the same black and yellow markings but is non-toxic. Two different molecular Müllerian tRNA mimicry rings exist in bacteria and eukaryotes, orientated around the 70S and 80S ribosome respectively (b) and (c). Prokaryotic release factor 1 and 2 (b), and eukaryotic release factor 1 (c), have parallel roles in translation but are not homologous. tRNA mimicry evolved independently in prokaryotes and eukaryotes. This observation implies that the original model molecule was tRNA, as opposed to a tRNA-like protein being the model for tRNA molecules themselves. In (c) a Batesian type tRNA mimic from Turnip Yellow Mosaic Virus (TYMV) is shown. The tRNA mimic is located 3’ of the virus genomic DNA, and enhances viral replication [43], benefitting the virus sender gene, but not the receiver (the host ribosome). In (d) the characteristic Chianti bottles containing wines from different Tuscan vineyards form a Müllerian mimicry ring, signaling to the consumer the type of wine. Fake Chianti mimics the bottle; this is Batesian type mimicry. For more detail on Chianti bottle mimicry see Supplementary Material Section 5. Sources for photos and 3D structures are in Supplementary Material

Likewise, all cellular tRNAs are signal co-mimics of each other, with the receiver the A-site of the ribosome. There are numerous additional tRNA-like co-mimics that are normal parts of the cell These include the yeast aspartyl-tRNA synthetase mRNA, *Escherichia coli* threonyl-tRNA synthetase mRNA, *E.coli* methionyl-tRNA synthetase mRNA (which all possess tRNA mimics on the mRNA leader; [35] [36] [37], the *Salmonella typhimurium his* operon [38], the mitochondrial Group I intron catalytic core [39], and ribosomal tRNA mimics (which are tRNA-like proteins that interact with the ribosome [40]). Several tRNA-like proteins interact with the ribosome, in the A-site. These molecules display Müllerian type mimicry, involving a cooperative relationship between the different tRNA shaped proteins and the ribosome, in both prokaryotes and eukaryotes (Figure 6b and c). These comprise molecular Müllerian mimicry rings, conferring a direct reward to the receiver, as opposed to the avoidance of harm in classical aposematic mimicry rings [41] (aposematism might be a special case of mimicry, with a pooled signal (‘I am toxic’) contrasting with the absence of a signal. In rewarding mimicry one might see a pooled signal contrasting with other signals, as in the wine bottles).

**Fig. 7:**
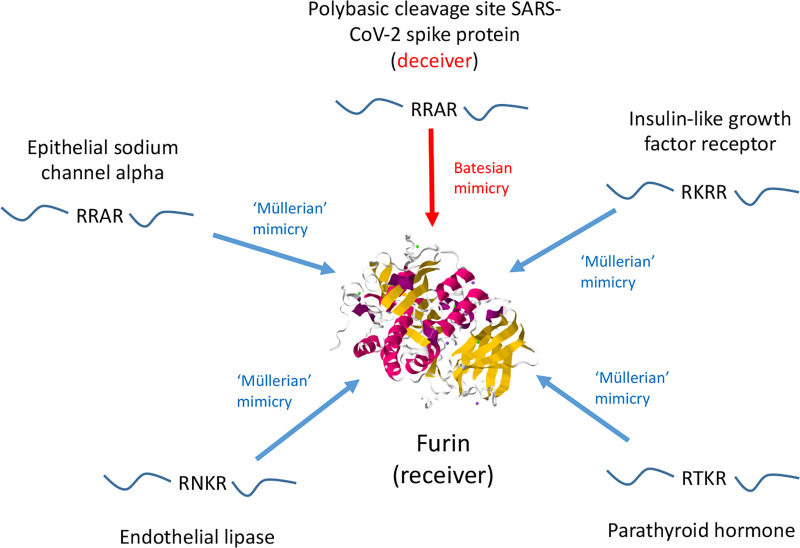
Invasion of a Müllerian molecular mimicry ring by SARS-CoV-2 spike protein polybasic cleavage site. Polybasic cleavage sites (PCSs) are found in an array of host proteins, and are cleaved by endogenous proteases, including furin [58]. The PCSs act as cooperative Müllerian co-mimics, forming a molecular mimicry ring; four examples are displayed in the figure [59] [60] [61] [62]. SARS-CoV-2 spike protein also contains a PCS, which leads to cleavage of the spike protein by endogenous proteases including furin, increasing the infectivity of the virus, and has directly contributed to the devastating effects of the COVID-19 pandemic [58]. The viral PCS deceives furin into cleaving it, constituting a Batesian mimic, which has invaded the PCS Müllerian molecular mimicry ring. This deceptive strategy is difficult to counteract pharmaceutically, because drugs that inhibit proteases from cleaving the viral PCS, will also inhibit the cleavage of endogenous PCSs that comprise the mimicry ring. The endogenous PCS signal is comprised of a short sequence, which is a cheap signal, meaning that it is easy to mimic. The large size of the mimicry ring means that the endogenous PCS signal is difficult to change, given the number of individual PCSs in endogenous proteins, and so an adversarial chase between endogenous and viral PCS signals is perhaps unlikely. These features may explain why the PCS is so commonly utilized by a wide range of microbial pathogens [63]. The ease of mimicry may also explain why it can arise in cancers [63], which despite utilizing a range of deceptive strategies, do not appear to make much use of Batesian mimicry, which can be costly to evolve. The structure of furin was obtained from the Protein Data Bank (5JXG).

In contrast, Batesian molecular mimicry involves a conflict of interest between sender and receiver genes. Batesian molecular mimics of mRNAs and tRNAs may be termed ‘deceiver’ mRNAs [33] and ‘deceiver’ tRNAs [14], respectively. Virus mRNAs are all effectively deceiver mRNAs, tricking the host translational apparatus into translating them regardless. Viruses also harbor a variety of ‘deceiver’ tRNAs, which trick the host translational apparatus by mimicking normal cellular tRNAs [14] [42] [43]. The fitness of the virus is enhanced, but at a cost to the host (an example is provided in Figure 6c). Numerous further parallels between molecular and organismal mimicry are discussed in Supplementary Material Section 3. The relevance of the signaling games perspective of molecular mimicry to the coronavirus SARS-CoV-2, and the severity of the COVID-19 pandemic, has not escaped authors’ notice. A key example is illustrated in Figure 7.

In early life, cellularization would have led to synchronization of sender and receiver gene replication, thus inducing common interest, and resulting in the alignment of their respective utilities. This process would have acted to promote cooperation, including Müllerian type molecular mimicry. In contrast, Batesian type molecular mimicry would have been disincentivized by the promotion of common interest. Conflicts of interest may still arise within the cell from selfish elements (i.e., insider threats in an intlligence organization), other forms of genetic conflict, and from external pathogens: this predicts the occurrence of molecular deception [14], which includes Batesian type molecular mimicry.

### 3.2. The role of molecular mimicry in COVID-19

The SARS-CoV-2 virus makes multiple uses of molecular mimicry in its efforts to exploit its human host, beginning its emergence via a zoonotic event from an earlier host, bat, which tolerates the virus in a quasi-Batesian mimicry ring. There follows a sample of some of the mimicry strategies that SARS-CoV-2 utilizes. i) Replication organelles, inside which the virus replicates [44], are a form of camouflage. ii) The addition of a cap-like structure onto the 5’end of viral mRNA by SARS-CoV-2 nsp16 [45], produces virus deceiver mRNAs, as discussed in Section 3.1. These are Batesian mimics of normal cellular mRNAs, which constitute a Müllerian mimicry ring, and is invaded by the viral deceiver mRNAs.

Further more, iii) Glycosylation of SARS-CoV-2 spike protein shields it from immune system surveillance [46]. Host glycans are acquired in the endoplasmic reticulum by several RNA viruses, and so glycosylated viral proteins are regarded as self by the immune system [47], while shielding the protein epitopes from recognition; this deceptive strategy constitutes a mix of Batesian and cue mimicry. iv) An elevated Ka/Ks in exposed regions of the spike protein is an indication of ancient adversarial chases between the virus and the immune system of mammalian host(s). This may be understood as an oscillation between recognition - evasion - recognition - evasion and so on, equivalent to the adversarial chase (0, 1) simulation, presented earlier in the Results section. Finally, v) the polybasic cleavage site (PCS) present in spike protein represents a Batesian molecular mimic, and playing a crucial role in the severity of the COVID-19 pandemic, described in more detail in Figure 7.

The most potent weapon in the human biotechnology-armamentarium against the Batesian deception of the virus is even cheaper molecular mimicry of the pathogen by vaccines. Vaccines may mimic different parts of a virus; its surface proteins, DNA or RNA. After administration of a vaccine, it deceives the human immune system into sensing that it is being attacked by a viable virus. This deception is costly to the vaccinated subject in the short term, as the vaccine itself does not bear any threat. However, the immune system retains a memory and so is pre-prepared for a (likely) future encounter with the real virus.

A virus may circumvent vaccination via antigenic drift, whereby virus epitopes mutate over time leading to novel immunogenic properties. This process will reduce the effectiveness of the vaccine, and is commonly encountered with flu vaccines, which must be generated on a yearly basis [48]. The response by the biomedical community to this, is to develop a new vaccine, which is a more accurate mimic of the newly evolved virus. Thus, a coevolutionary chase is joined, between vaccine and virus, that bears similarity to the oscillatory effect displayed in Figure 4.

Better knowledge of the deceptive strategies of SARS-CoV-2 will help to inform vaccine design. Particularly, a better understanding of decoy (non-neutralizing) epitopes will help in the rational design of vaccines using peptides. Decoy epitopes result in the production of non-neutralizing antibodies by the immune system, and can lead to antibody dependent enhancement (ADE). This phenomenon occurs when decoy epitopes bind to non-neutralizing antibodies which facilitate the entry of the virus into the host cell [49]. Decoy epitopes result in a reduction of efficiency of vaccines, by diverting immune resources away from the recognition of neutralizing epitopes [50], and by potentially causing ADE [49].

The prediction of decoy epitopes from virus protein sequences has been little studied. Understanding the evolutionary dynamics of decoy epitopes may allow their more precise identification. A key question to be answered is whether they are adaptive; if so then they may be better understood as Batesian mimics of neutralizing epitopes. In this scenario, the decoy epitope is deliberately exposed to the immune system, rather than being shielded by glycans, in order to divert antibodies from the neutralizing epitopes. Identification of decoy epitopes will allow the design of vaccines that circumvent such epitopes, thus sharpening the immune response to the vaccine.

Anti-viral drugs are also typically molecular mimics. For example, remdesivir is a molecular mimic of ribonucleotides. The drug represents a deceptive molecular signal, luring the virus replicase into binding to it. The response of virus over time is to develop drug resistance, by ceasing to bind the drug; it has ‘learned’ that the drug is deceptive.

The virus may develop resistance more slowly to some drugs than to others. We take as our inspiration the model of Polybasic Cleavage Sites (PCS) mimicry displayed in Figure 7, where the Müllerian molecular mimicry ring of PCS signals present in endogenous proteins constrains the signal sequence from diverging, in response to PCS mimicry by SARS-CoV-2 spike protein. Likewise, if an anti-viral drug invades a molecular mimicry ring formed by the viral protein and its canonical substrates, then the viral protein may not easily change its specificity and develop drug resistance. This stability sustains because it is constrained by the need to bind several canonical substrates, which are similar in structure.

### 3.3. Cellularization-like processes and the evolution of cooperation

Analogous processes to cellularization, which involve the alignment of utilities thus promoting cooperation, abound at higher levels of biological organization (Table 2). In human society, the formation of trading blocs and religious denominations, tribalism, and groupings propelled by homophilic and other group splitting processes, including some considered harmful such as balkanisation, may also be considered forms of cellularization.

While such cellularizations are relatively stable, nonetheless, they can be sporadically destabilized by environmental changes that alter relationships among players (e.g., trust as measured by correlation of encounters) – e.g., social-distancing to mitigate a viral pandemic spread. The resulting cascade of disruptions among employeremployee relationships give rise to a Shumpeter’s gale, a creative destruction dynamic, analogous to a coevolutionary chase, resulting in recellularizations, which may have to be coordinated carefully with artificial and temporary shifts in the utility functions: e.g., unemployment benefits, bail-outs, basic incomes, etc., but may also lead to extensive mimicry. The dynamics of recellularization in the presence of Batesian mimicry may lead to *L*, *U*, *V* or *W* shaped economic recoveries and warrant further investigations in the macro-economic contexts.

To break the cycle of constant invasion (*W*-shaped recovery), we speculate that nature and games must have a mechanism for stabilizing cellularization, this procedure strongly solidifies a tighter alignment bond between utilities of cooperative components and preserves the signaling system while also offering security recourse or a means for its protection often by costly signaling, or increasing the price for mimicry and thus disadvantaging invasive Batesian types.

Also we speculate that the mixed mode cycle completed by *transition A, B, C* in Figure 5 may afford evolution with a duty cycle. The return to a babbling state can allow retrial of various cooperative mimicry component combinations.

A particularly interesting socio-technological question comes up in the context of social-distancing aspects of pandemic measures and its effects on economic relations – frequently mischaracterized in terms of lives-vs-livelihood trade-offs. Social distancing has led to novel applications of digital communications, automation, artificial intelligence and *in silico* simulations and poses interesting questions about restructuring dynamics for our macro-socio-economic worlds (e.g., guitar-string model and whether the recovery would be *V* or *W*-shaped). An important question posed in the context is the role likely to be played by the currently available (unexplainable) AI technologies and the “pandemonia of imitations” it may give rise to. Here, a mass of imitations may be used to increase the probability of a successful invasion of a Müllerian mimicry ring.

Though creativity, intelligence and problem solving play many important roles in modern economic relations, they have been difficult to formalize. For instance, computability has a widely-accepted model in terms of Church-Turing thesis, Turingreducibility and Turing-universality, but as a consequence of these, it remains impossible to define computers’ (classical or otherwise) general problem solving capability necessary for automation of economic tasks: including estimating whether a particular task (specified in a contract) may be considered reasonably completed – the classical Halting Problem. In fact given two programs: one genuine and other (presumably) imitative, there can be no decision procedure to determine if they are Turing equivalent. These statements have deep implications on how we may wish to define Artificial Intelligence and its potential role in economic infrastructure.

The solution Turing suggested was in terms of *mimicry* in Information-Asymmetric Signaling games: involving a certain set of sender agents, some of which will have the type Oracles (e.g., humans) and the others of the type Imitators (e.g., models). The senders send certain signals (e.g., conversational statements in English) to receivers (e.g., humans) who must act by responding to Oracles, but ignoring Imitators. Such a game may be called an Imitation Game and the receivers test a Turing Test. As a signaling game the classical Imitation Game and its extension both have Nash Equilibria: some trivial such as Babbling or Pooling but others far more relevant to present discussion: namely, separating. A natural way to define Artificial Intelligence would be in terms of Imitators ability to achieve a reasonably informative and stable pooling (non-separating) Nash Equilibrium when introduced into a society of human Oracles.

One may propose a solution to the economic cellularization problem, which involves extending the economic system to include additional non-strategic agents: namely, *Recommenders* and *Verifiers*. These AI agents will have no explicit utilities to optimize (or even, satisfice) other than those described in terms of winning (or losing) certain tokens. An individual (homo-economicus) may envision organizing one’s recommenders and verifiers not just playing imitation games in various disjoint circles of one’s socio-economic lives, but also forming stable Müllerian mimicry rings to restore one’s relationship with others in a rational utility-optimizing manner. Engineering these AI-augmented humans would be the core problem for AI: The ultimate Turing Test for the set of intertwined imitation games we call a modern civic society and its markets, falling and rising as motioned by an invisible hand.

## Supplementary Material

1

## Figures and Tables

**Table 1:** Mimicry at multiple levels Mimicry operates at all levels of biological organization. Listed in the table are a number of illustrative examples of the major characteristics of mimicry

Feature of mimicry	Molecular level	Organismal level	Societal level
Batesian mimicry	Viral tRNA mimics imitate host tRNAs (Figure 6c)	The beetle *Clytus arietis* is a Batesian mimic of bees and wasps (Figure 6a)	Asymptomatic patients that go undetected in contact tracing analysis, Phishing attacks. Psychopaths display affective mimicry (mimicry of emotions) [7]
Müllerian mimicry	Cellular tRNA isoacceptors are co-mimics, as are the common 5’leaders and 3’ polyA tails of mRNAs for different genes (Section 3.1)	Bees and wasps are Müllerian co-mimics, sharing a common signal of a black body with yellow stripes	The Anonymous hacktivist collective is a manifestation of cooperative mimicry, as are voluntary COVID-19 research teams
Mimicry ring	tRNA molecular mimicry ring comprised of tRNAs, and tRNA-like mimics where the receiver is the ribosome (Figure 6b and c)	Multiple bee and wasp species form a mimicry ring, where the receiver is a potential predator (Figure 6a)	The Silkroad vendor website, used to sell illegal products and services, has spawned offspring after it was shutdown, that mimic the appearance and functionality of the original website, including the webmaster, Dread Pirate Roberts. Pirate flags themselves constitute a mimicry ring, discussed in Supplementary Material Section 2
Cue mimicry	Cancers can blend into the host tissue, by a variety of mechanisms such as surface antigen masking by sialic acids [8], down-regulation of MHC Class I expression [9] and truncation of oligosaccharides on cell surface proteins [10]. Devil facial tumor disease 1 (DFT1) is a contagious cancer in Tasmanian Devils, which blends into the somatic background, facilitated by loss of MHC Class I molecules [11]	Spiders and chameleons can blend into their respective backgrounds	Zero day vulnerabilities are very difficult to detect as they blend into the source code. Some may occur accidentally, others may be deliberately introduced. A form of cue mimicry in cyberspace is protocol spoofing, which describes a means of concealing a communication within another type of communication so as to avoid its detection by a government or service provider that could potentially snoop. Additional examples include Tor’s anonymization routing, anti-censorship and free speech technologies. A recent example is found with protesters in Hong Kong, aware that officials and police use biometrics such as facial identification, have utilized face masks to protect their anonymity. [12]
Complexity and stability	Simple, stable, close to optimal	Moderately complex and stable, but requires costly mechanisms that might cause species extinctions [13]	Highly complex, involving multiple institutions with complex checks and balances. Stability is poorly understood.
Costly (honest) signal	The tertiary structure of proteins represents a costly signal difficult to mimic [14]	Animals display costly signals such as sexual ornamentation [3]	Proof of work by bitcoin miners is a costly signal. Other examples of costly signaling include risk taking by health workers, conspicuous consumption [15], educational attainment [16], and potentially cognitive capacity [17]

**Table 2:** Game payoff matrix describes outcomes between predator and various types of organisms. Notice that co-mimics are no different than model and are considered Müllerian, while mimic is Batesian. An example game parameterization used throughout the results section is: *α* = 0.4, *β* = 1.0, *κ* = 0.1, *ρ* = 0.1, and *ν* = 1.2.

payoff (predator, prey) encounter				

predator action	model	mimic	co-mimic-A	co-mimic-B

avoid	(*α*, *β*)	(*α*, *β*)	(*α*, *β*)	(*α*, *β*)
consume	(*κ*, *ρ*)	(*ν*, *ρ*)	(*κ*, *ρ*)	(*κ*, *ρ*)

**Table 3:** Cellularization-like processes at multiple levels Cellularization-like processes promote cooperation and involve the alignment of utilities. This can occur at different levels of biotic complexity, ranging from biomolecules to whole organisms and then to human society, and ultimately supra-national organizations.

Level	Type of cellularization	Examples	Type of deceptive mimicry it is susceptible to
Biomolecular	Between the first genetic units of inheritance	The first genes are unknown but interactions between their expression products would have constituted the first biomolecular signaling games. Deceptive signaling would be minimized by common interest, induced by synchronized replication resulting from cellularization	The first genes would have been susceptible to deceptive molecular mimicry
	Protein translation	Between mRNAs and the ribosome	‘Deceiver’ tRNAs (’Batesian’ tRNA mimics) and ‘Deceiver’ mRNAs (such as virus mRNAs, Section 3.1)
Genomic	Between the genes within the genome	Synchronized replication of all genes in a genome	Selfish elements may replicate at the expense of the host genome; there is evidence that they engage in Batesian molecular mimicry [14]
Cellular	Endosymbiosis	Eukaryosis involved the acquisition of a mitochondrion within the proto-eukaryotic cell [51]	Intracellular pathogens persist by a variety of evasive mechanisms, including molecular mimicry [52]
Organismal	Eusociality	Ants	Ant social parasitism often involves deceptive cuticular hydrocarbon signals [53]
	Flocking, herding and swarming	Bird flocks	In a mixed species flock, fork-tailed drongos will emit false alarms, causing other species to drop their prey [54]
Societal	Tribalism	Tribal groupings are found all over the world, and appear to be an ancient human behavior [55]	Cooperation between non-kin in tribal societies is susceptible to freeriders, which may be controlled by sanctioning [56]
	Religions	Religions, and their sects, down to local groupings such as churches, synagogues and temples	False prophets and some forms of virtue signaling
	States	These may be statelets, autonomous regions, nation or federal states	Freeloading behavior via avoidance of paying tax, which often involves deceptive mimicry (tax represents the fiscal contract between citizen and state [57])
	Trading blocs	Examples include the European Union (EU) and the Association of Southeast Asian Nations (ASEAN)	Counterfeiting (Batesian mimicry) and smuggling (cue mimicry)

## Footnotes

1 The alternative decision function, to target features for predation is equivalently represented with *δ* > 1. The even simpler half space model is achieved when *δ* = 1. The important modeling aspect is that the decision function spans multiple types with a single avoidance feature, this constraint is intended to represent realistic trade-offs between adaptability, quality and cost in evolving complex decision making mechanisms.

2 This population structure closely resembles the classical mimicry scenario with the toxic type as *model*, non-toxic type as *mimic*, and predator *as dupe.*
