# Supplementary material for: HOW SIGNALING GAMES EXPLAIN MIMICRY AT MANY LEVELS: FROM VIRAL EPIDEMIOLOGY TO HUMAN SOCIOLOGY
Source: Res Sq. 2020 Aug 6:rs.3.rs-51959. Preprint. [Version 1] doi: 10.21203/rs.3.rs-51959/v1 (PMC7418725; doi:10.21203/rs.3.rs-51959/v1)
Supplement: 1 [file NIHPPrs51959v1-supplement-1.pdf]

4. Supplementary Material.

Supplementary Material 1: Methods.

Geometry and visualization of high dimensional signals, cues, and markings. Signal space To model signals for organisms we use a high dimensional vector space as points in $\Gamma = [-1, 1]^D$, for D in the range $[5, 1000]$. The components could generally translate to specific features of morphology or behavior. The geometry of symmetric sets (such as Γ) in high dimensional vector spaces exhibit interesting orthogonality properties stated statistically: As D increases, two random vectors form Γ are increasingly likely to be nearly orthogonal. More precisely, for any $0 < \epsilon << 1$:

$$\text{Prob}\left(\left|\frac{\langle x_1, x_2\rangle}{||x_1||||x_2||}\right| < \epsilon\right) \to 1 \text{ as } D \to \infty$$

This convergence can be strongly sensed in vector spaces with dimension D as low as in $[5, 15]$. Noting that the range of the $\frac{\langle x_1, x_2\rangle}{||x_1||||x_2||}$ is bounded in $[-1, 1]$, the cosine distance function, commonly defined as:

$$d_{\cos}(x_1, x_2) = 1 - \frac{\langle x_1, x_2\rangle}{||x_1||||x_2||}$$

Will have range $[0, 2]$ with concentration of values at one for random vectors $x_1, x_2 \in \Gamma$.

We use these facts to create a model for predator decision making. In our model the receiver (predator) maintains a high dimensional vector as a mechanism to evaluate if it should perform an action (avoid predation or otherwise). The organism sender $\mathcal{S}$ will present a signal vector $x \in \Gamma$. The receiver $\mathcal{R}$ will utilize a decision vector $y \in \Gamma$ (fixed over the organisms life span but amenable to mutation in successive generations), and a threshold τ (likewise fixed but amenable to mutation) to decide whether to consume $\mathcal{S}$ if $d_{\cos}(x, y) < \tau$ or not (otherwise). The decision function generalizes a half-plane model where $\tau = 1$.

Visualizing Strategies: To visualize these high-dimensional signals we use *star glyphs*. Star glyphs provide a means to visualize high dimensional data in a way that is relatively neutral to recognition features which would otherwise be cognitively engaged. Below in figure S1 we illustrate an examples.

To glimpse the effects of mutating a signal in high dimensional space, where random vectors are likely found to be orthogonal (see S2 (a)), We can still get an idea of the displacements our mutation operation makes by fixing base vector $X \in \Gamma = [-1, 1]^7$, and showing the effects of weighted averages with other random vectors. In figure S2 (b) we illustrate a few samples which are are scaled in increments and their associated distance from the base vector. Holding a signal $X \in \Gamma$ constant we generate a sequence of displaced signals: $Y_k = \lambda_k W_k + (1 - \lambda_k)X$ for W_k drawn uniformly for each $k \in \{1, 2, \ldots 20\}$ (twenty distinct samples) and $\lambda_k = k \times \frac{1}{20}$. We plot the resulting cosine distance from X to vectors Y_k as a function of k. This illustrates both the trend and the variation inherent to the mutation operation.

Measuring Population Dynamics and Identifying Mimicry:. We expect that signal mimicry will emerge due to the increased utility it offers. Noting that signal mimicry need not imply signals be fixed and constant, a suitable measure should account for dynamic signal evolution so long as the co-evolutionary constraints are clearly discerned. To capture mimicry we design statistical measures referenced from the evolving predator strategies.

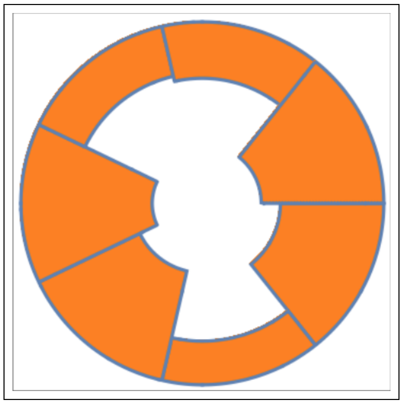

Fig. S1: Star Glyphs provide a useful means to visualize organism signals or the multi-dimensional sensory cues that they send to a receiver predators. The same star glyphs can be used to represent what features a predator may avoid. The star glyph is a set of wedges which indicate the weight assigned to each component, these glphys represent vectors from $\Gamma = [-1, 1]^7$, and starting from the positive three o'clock position and progressing in a counterclockwise rotation around the circle, seven weights can be seen as the radius at which the wedge starts, the more of the wedge that is missing the more weight is placed on that component.

With these measures, *signal mimicry* among types will be characterized by: 1) low intra-type signal variation and 2) low extra-type signal variation. Taken together these indicate co-evolution to the benefit of at least one type. For example, in the Müllerian ring, when several types have similar markings, the signaling would be characterized by low extra-type signal variation. Low intra-signal variation occurs if the signaling traits are conserved.

Boosting Distribution:. Fixing the time step and the type, let ϕ_s for $s \in \Gamma$, be the performance measure attained by strategies implemented during that time step. Letting $\phi_* = \min(\{\phi_s\})$ and $\phi^* = \max(\{\phi_s\})$ we can safely transfer the performance measures to the interval $[0, 1]$ as the limit of fractional transformation:

$$V_s^\xi = \lim_{\eta \to 0^+} \frac{\phi_s + (\xi - \phi_*)}{\phi^* - (\phi_* - \eta)}$$

The term η simply prevents division by zero, and the term ξ is a *statistical shrinkage* term used as a model parameter that helps to distort global information available to agents when they re-select a strategy.

We describe the probability that $s \in \Gamma$ switches over to use the strategy $s' \in \Gamma$ as:

$$\Phi_{s'} := V_{s'}^\xi / \sum_{s \in S} V_k^\xi$$

. In our simulations, noting that all rewards are positive (albeit greatly reduced when prey is consumed), we fix $\xi = 0$.

Descriptions of mimicry modes. Batesian, Müllerian, Deceptive Cue, Cooperative Cue mimicry

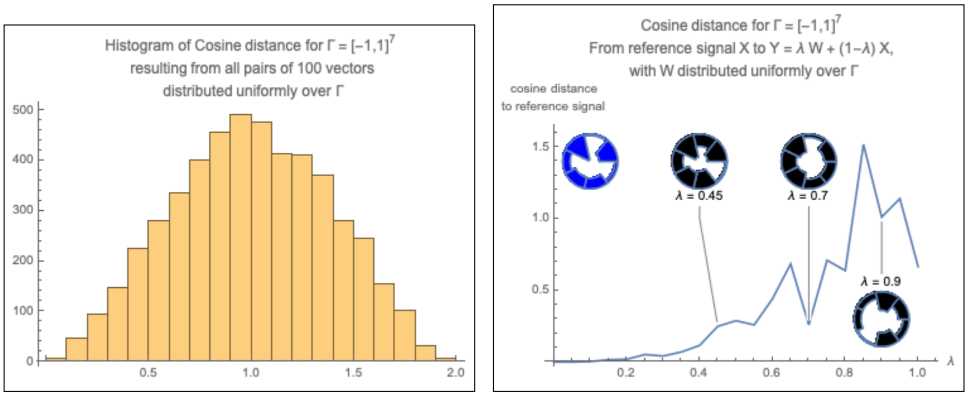

(a) (b)

Fig. S2: Geometry of signals space, Although statistically most random pairs of signals from $\Gamma = [-1, 1]^7$ will be near orthogonal by having cosine distance near 1.0 such as indicated in the histogram in (a). In (b) will illustrate a few sampled random scaled displacements of a base vector (blue) look like as well as their cosine distance. Holding a signal $X \in \Gamma$ constant we generate a sequence of signals $Y_k = \lambda_k W_k + (1 - \lambda_k)X$ for W_k drawn uniformly random over Γ and $\lambda_k = k \times \frac{1}{20}$. The cosine distance is plotted as a function of k and a few star glyph visualizations are illustrated. This gives a sense of the high dimensional random mutation operations in the evolutionary game.

Supplementary Material 2: Pirate flags as Müllerian mimicry. The 17th-19th century pirate flag, the Jolly Roger, appears to have constituted a shared signal of toxicity to those crews that resisted boarding. There were a range of such flags with common elements, such as a black background, with a white skull and other objects such as weapons, bones and bleeding hearts. An interesting example of Batesian type mimicry in this context is the use of a legitimate flag by pirates in order to get close to a ship, and then raise the pirate flag when close enough to board ('showing one's true colors').

There were different gradations of pirates. A true pirate was an outlaw, who would be executed on capture. A 'privateer' was a legitimized form of piracy, at least on the part of the licensing nation, and so expected to give quarter when encountering intransigent ships. Pirates varied in ferocity (ie. toxicity), and so a fearsome reputation could be an asset. Consistent with this, some pirates took efforts to enhance their notoriety. Blackbeard presents a well known example, by braiding and growing his beard long and attaching lit fuses to his hat in battle [64], he was also reported to have taken periodic potshots at his crew, reasoning that "if he did not now and then kill one of them, they would forget who he was" [64]. An early pirate history recounted that "In the Commonwealth of Pyrates, he who goes the greatest Length of Wickedness, is looked upon with a kind of Envy amongst them..." [64].

Lastly, mixed (quasi-Batesian) mimicry would occur if a pirate imitated a more famous pirate, in order to more easily board merchant ships. A potential example of this appears to be Francis Spriggs, who is reported to have flown a Jolly Roger identical that of pirate Captain Low's, from whom he had deserted, "...a white Skeliton in the Middle of it, with a Dart in one Hand striking a bleeding Heart, and in the other, an

Hour-Glass..." [64]. In this scenario the pirate flag would be partially honest (allowing identification as a pirate), and partly deceptive (promoting identification with a more famous and presumably fearsome pirate), and so constitutes mixed mimicry between Müllerian and Batesian type mimicry.

Supplementary Material 3: Further parallels between molecular and organismal mimicry. Given that natural selection operates at both the organismal and molecular levels, one might expect to observe numerous parallels between organismal and molecular mimicry. For example, at the organismal level the frequency of the model is an influence on the development of Batesian mimics: the more common the model, the more likely mimics are to arise [65]. In a potential parallel, there is a high concentration of tRNA in the cell because protein translation is a major cellular function, so the model tRNA molecule is present at high frequency in all living organisms, and so this may have influenced the widespread development of Batesian tRNA mimics by viruses.

In organismal Müllerian mimicry, the mimic is expected to evolve to become more similar in appearance to a model organism, in a process known as advergence [41]. The two step hypothesis proposes that a potential co-mimic first undergoes a mutation that causes a major change in phenotype, becoming more similar to the model. Then, if this is advantageous further mutations lead to an increase in similarity to the model ([66], a summary). In molecular Müllerian type mimicry, a similar process might be expected to occur. Consistent with this, in the case of EF-P, a cellular tRNA mimic, it appears that the protein has evolved over time to become more tRNA-like, in a process of advergence [67]. Presumably, tRNAs predate the various other tRNA co-mimics found within the cell, and so comprises the model. A potential example of Batesian type molecular mimicry resulting from intragenomic conflict is that of the Mauriceville mitochondrial retroplasmid of *Neurospora crassa*, that uses a tRNA-like structure at the 3‘ end of the plasmid transcript to initiate cDNA synthesis [68]. Examples of organismal and molecular Müllerian are illustrated in Figure S3.

'Rewarding' mimicry has been proposed as an additional category of mimicry [5]. A rare example is provided by floral mimicry, where there is evidence that two plant species *Turnera sidoides* ssp. *pinnatifida* (Turneraceae) and *Sphaeralcea cordobensis* (Malvaceae) share a common signal, the shape and color of the flowers [69]. The sharing of a common signal between two flower species leads to more effective signaling to the pollinator, because the frequency with which the pollinator encounters the signal is increased, which means that the association between signal and reward (the pollen / nectar) is more efficiently learnt by the pollinator.

While organismal behavior is more complex than macromolecular behavior, some parallels can be drawn between the two. Thus, while 'receiver psychology' at the organismal level refers to the receivers ability to detect, discriminate and remember a signal [32], an equivalent can be found at the molecular level in the aptitude of a receiver macromolecule to distinguish a particular macromolecular signal. This is dictated by its biochemical binding affinity, which reflects the strength and specificity of binding between a macromolecular signal and receiver macromolecule, quantified by a binding constant such as the Michaelis-Menten constant (Km). Thus, a macromolecular mimic should display a similar binding affinity and specificity with a receiver macromolecule, to that of the model macromolecular signal.

At the organismal level, selection on the model to evolve differences from the Batesian mimic is expected, so that the dupe can better distinguish the model, as opposed to the mimic [70]. With cellular tRNA models, their conformation is tightly

constrained by their role within the ribosome. However, we propose that the large number of tRNA base modifications [71] may have evolved so that the host organism can better distinguish viral deceiver tRNAs, which are deficient in modifications. If tRNAs with base modifications are harder to mimic, then these constitute a 'costly' signal.

At the organismal level, in order for the potential dupe to detect Batesian mimics, a variety of scanning and surveillance mechanisms have evolved [19]. Likewise, we expect molecular scanning mechanisms to have evolved to detect Batesian tRNA mimicry. These might include translational proofreading mechanisms (by aminoacyl-tRNA synthetases, and the ribosome), however these are likely to have evolved primarily for the important role of ensuring translational fidelity. Information asymmetry exists between the pathogen (or selfish element), and the host organism, hence there should be a selection pressure to evolve scanning mechanisms that can ameliorate this asymmetry. Consistent with this, in the case of the mimicry of eukaryotic initiation factor 2α (eIF2α) by viral eIF2α mimics, there is evidence for a selective pressure on the macromolecular receiver, protein kinase R, to evolve more effective recognition of eIF2α [72].

In a parallel, antimicrobial drugs often act as Batesian molecular mimics of biological macromolecules. For example, the antibiotic puromycin is an organic compound synthesized by *Streptomyces alboniger* that mimics tRNA [73], inhibiting bacterial translation. This process involves Batesian type tRNA mimicry, on the behalf of the physician(the sender), with the bacteria (the receiver), who experiences a drop in utility. Microbial drug resistance can be understood as the result of a selective pressure for more efficient detection by the pathogen of molecular mimicry.

In cue mimicry, there is only one sender, which mimics a cue, sending the mimicry signal to one or more receivers. This may be considered a modification of the partial pooling equilibrium, where the receiver may receive both a signal and cue, both of which will elicit the same action.

Rather than relying on increasingly costly signals, an additional strategy to detect mimicry might be to harness multiple receivers to determine signal veracity (which could be summarized by the phrase 'you can fool some of the receivers some of the time, but not all of the receivers all of the time'). In contrast, at the organismal level, multiple receivers have been tentatively linked with the occurrence of imperfect (low fidelity) mimicry. This is hypothesized to result from differences in sensory perception amongst receivers [74].

Lastly, learning a visual warning signal is important in the evolution of Müllerian and Batesian mimicry [41] [75]. While learning is a complex behavior deriving from neurological processes, a simpler but equivalent process may be found at the macromolecular level given that learning can be viewed as a process that a population undergoes over generations, as the strategies exhibited by individual members of the population change as a result of selection [18].

Supplementary Material 4: Mixed mimicry. Butterflies of weak toxicity show mixed mimicry, also termed 'quasi-Batesian' mimicry [77]. There is evidence that this allows them to freeride on the signal value of highly toxic species [78]. The prediction has been made that quasi-Batesian mimics might have a stronger selection pressure for signal accuracy, than their Müllerian co-mimics, and so the quality of the mimicry is superior [78]. This then suggests a manner of detecting quasi-Batesian mimics, by their greater quality signal (the common phrases 'too good to be true' or 'holier than thou' suggest that humans may be attuned to detect such strategies).

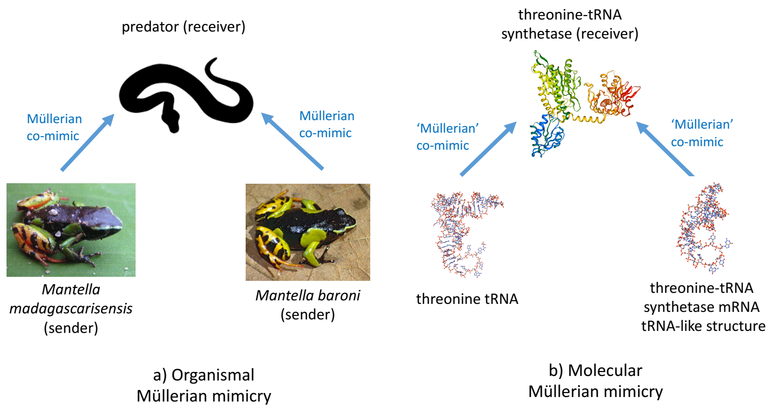

Fig. S3: Organismal and molecular Müllerian mimicry. a) shows an example of organismal Müllerian mimicry between two species of poisonous frogs from Madagascar [76]; (b) shows molecular Müllerian mimicry, illustrated by an example of cellular tRNA mimicry, where the mRNA of *E.coli* threonyl-tRNA synthetase (thrRS) mimics a tRNA structure (apparently in a negative feedback loop, [35]). ThrRS-mRNA and thr-tRNA are co-mimics, both interacting with thrRS, the common receiver. Here, the genes for thr-tRNA and thrRS are the senders, while the signals are the three dimensional structure of thr-tRNA, and the tRNA-like structure in the leader of thrRS mRNA. The two senders, and their respective signals, are co-mimics. Information regarding three dimensional structures and photo sources is located in Supplementary Materials.

A mimicry spectrum has been proposed to exist from those organisms that display purely Müllerian mimicry to those that are purely Batesian mimics [79]. This is because different species can vary in their levels of toxicity, and so those with lower toxicity are expected to be less cooperative, and their mimicry more Batesian, as a consequence. Signaling game theory would predict that mixed mimicry would occur when there is partial common interest between sender and receiver. The mimicry spectrum ranges from opposed interests (Batesian), to perfect common interest (Müllerian). At the molecular level, mixed mimicry would be expected to occur in cases of intragenomic conflict, given that interests are not perfectly aligned between differing components of the genome. Likewise, some cases may occur where pathogenic microbes do not have completely opposed interests to the host, and there is a degree of common interest, which concords with the Tradeoff Hypothesis [80].

Mixed mimicry might also be expected to occur within the 'parasitism - mutualism continuum'. This refers to the transition of microbes from pathogenicity (with opposed interests to the host) to mutualism (with perfect common interest with the host) that occurs over evolutionary time [81]. Between the two extremes of pathogenicity and mutualism there exist states of partial common interest. Here, a mixture of Batesian and Müllerian molecular mimicry is expected to be encountered. Thus, in

the case of viruses, deceiver mRNAs might be expected to bring some benefit to the host, if there is a cooperative component to the virus infection. In this case, the characteristic sequence and structure of the cellular mRNAs 5' leaders and 3' polyA tails can be considered as cooperative co-mimicry signals. Virus encoded mRNAs may mimic these signals [82], but if some in some way the virus contributes to organismal fitness [83], this might present an example of quasi-Batesian type molecular mimicry.

Supplementary Material 5: An economic Müllerian mimicry ring. Many Chiantis use a distinct bottle with a basket (the *'fiasco toscano'*). Originally, this had the practical purpose of protecting the bottles during transport [84], and so was not intended as a signal, instead constituting a 'cue', an observable feature, not intended as a signal. The original purpose of the basket has been superseded by the use of stronger bottles, but the *fiasco toscano* has been retained by some producers, acting as an identity signal [85]. This presumably benefits the consumer as they can more easily identify a Chianti wine, due to their common signal, the shape of the bottle and the basket. Therefore, this appears to be an example of a rewarding Müllerian type mimicry ring, as both multiple senders, the vineyards which act as co-mimics, and the receiver, the customer, benefit (Figure 6b).

Chianti is one of the most heavily counterfeited of all wines [85], to the extent that there is a webservice dedicated to verifying the identity of a bottle of Chianti (www.chianticlassico.com/en/wine/traceability/). The counterfeiting constitutes Batesian type mimicry, causing harm to both the deceived customer, and producers of genuine Chianti. Interestingly, in order to maintain honest signaling, the signal (the basket) itself has evolved over time to reduce counterfeiting. For example, the basket was reduced to shoulder height in order to accommodate an identifying lead seal (a 'costly signal') in the neck of the bottle.

Supplementary Material 6: Image sources. Photos were obtained from the Encylopaedia of Life: Figure 3(a), *Mantella madagascarisensis* and *Mantella baroni* (Encylopaedia of Life); Figure 6, *Sceliphron laetum* (potter wasps); *Anthidiellum notatum* (leafcutter bees); *Polistes exclamans* (paper wasps); *Philanthus traingulum* (beewolf); (bumble bees); (carpenter bees); (social wasps); (hornets); (eusocial bees); (social wasps); *Clytus arietis* (wasp mimicking beetle);Figure 5, images of Chianti bottles were obtained from the manufacturers websites: www.rufino.com (Ruffino winery), www.davinciwine.com (Leonardo da Vinci winery), www.opici.com (Opici wines), www.sensivini.com (Sensi winery)

Three dimensional molecular structures were obtained from the Protein Data Bank, the respective PDB identifiers are: Figure 6b) *E.coli* thrRS-mRNA (1KOG), *E.coli* thr-tRNA (1QFS) and *E.coli* thrRS (1QFS); Figure 4(a) the *E.coli* 70S ribosome (4V4A), *E.coli* phe-tRNA (3LOU), *Streptococcus mutans* release factor 1 (1ZBT), *E.coli* release factor 2 (1GQE), *Thermus thermophilus* ribosome recycling factor (1EH1), *Acinetobacter baumannii* elongation factor P (5J3B), *T.thermophilus* elongation factor G (1ELO), *E.coli* Etta translational throttle A (3J5S), *T.thermophilus* tmRNA-SmpB complex (2CZJ); Figure 6b) the human 80S ribosome (4UG0), *Saccharomyces cerevisiae* met-tRNA (1YFG), TYMV tRNA-like structure (4P5J), human eukaryotic release factor 1 (1DT9), *S.cerevisiae* Dom34 ribosome rescue factor (2VGN), and *S.cerevisiae* eukaryotic elongation factor 2 (1NOV).

Simulation Studies. Using the methodology outlined above, we construct a set of systems characterized by differing population structures. While most parameters of the system are kept fixed, the differing numbering of Müllerian and Batesian types

provides insights on how mimicry forms, becomes stable, and unstable so that populations may adapting signaling strategies which meet their *protective* and *metabolic* requirements. Specifically for each system we perform simulations and apply measures to their simulated histories in order to evaluate mimicry.

To organize the study we introduce a structure index. To keep the study as simple as possible we will fix the number of predator types to be 1. Therefore the index will identify the number of Müllerian types and number of Batesian types as tuple: (M, B). Recall that we focus on the Predator's avoidance of toxic organisms, so the Müllerian types are toxic working with predator to learn avoidance. The Batesian types are non-toxic, and use deceptive or mimetic strategies. For each type (including the predator) will will fix the population size to be $N = 100$.

The systems are then ordered as a lattice in figure S4. We will make reference to

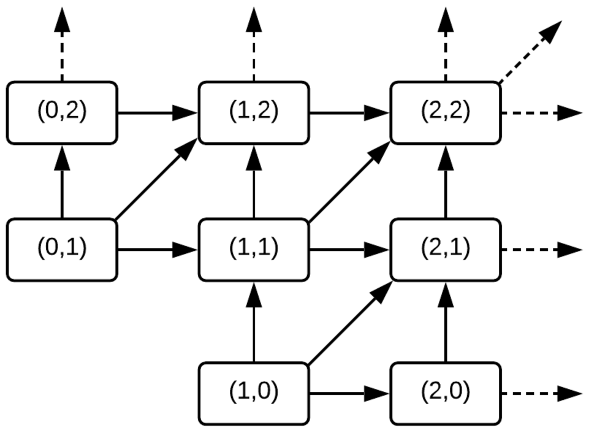

Fig. S4: Simulation study overview. We perform simulations for a variety of systems indexed by (M, B), as the number of toxic and non-toxic types that can be encountered. An environment could be described by a particular node, directed arrows indicate how the addition of type may transition the environment.

the index while illustrating results.

Simulation experiments.. Below we outline simulation experiments by stating the hypothesis that are considered and tested. Results are illustrated and discussed.

RL: System $(1, 0)$ expresses signal locking a type of reinforced learning. It has one toxic type and one predator type, thus it is in the common interest to emerge an honest signaling convention. This experiment will test that types learn a convention of avoidance. We hypothesis that once learned, the signal convention is stable, a condition we call *signal locking*. Even if the convention is stable, the signal itself will likely drift.

AD: System $(0, 1)$ expresses adversarial dynamics and learning. It has one non-toxic type and one predator type whose goals are in opposition. The non-toxic type may exploit the predators avoidance for its protection by a Batesian signaling strategy. The predator must update avoidance to secure its metabolic

requirements. The results are used to evaluate if this dual optimization seeking opposing goals is expressed.

MR: System $(2, 0)$ has two species of toxic prey and a single species of predator. We test that predator and both non-toxic types can lock signals to secure a ring of honest signaling. The result will indicate the stability and duration to Müllerian rings.

MM: System $(1, 1)$ is mixed mode composing system $(1, 0)$ and $(0, 1)$. It has one non-toxic, one toxic and one predator type. This experiment evaluates the dynamics of the classical model (honest sender), mimic (dishonest sender), and dupe (receiver) scenario. In particular this examines the role of Batesian mimicry; in particular, if a signaling convention among the predator and toxic types emerge then will it be evolutionary stable to the invasion of deceptive signal (arising from a nourishing species mimicking a toxic one).

AD2: System $(0, 2)$ expresses a double adversarial learning scenario. It has two non-toxic types and one predator type. The goals for the non-toxic type are in opposition to the predator. The receiver must update its avoidance parameters to prevent loss.

MRA: System $(2, 1)$ expresses a Müllerian Ring that can be invaded by Batesian type. It has two toxic, one non-toxic, and one predator type. Results will evaluate dynamics leading to: ring formation, stability, loss and reformation.

S12: System $(1, 2)$ expresses mostly adversarial dynamics inherited from system $(0, 2)$, but includes the component system $(1, 0)$ which offers the receivers a minor reprieve if an honest signaling convention can be achieved. Results help to evaluate the mixed mode dynamics and the destabilizing role of multiple Batesian types.

MM2: System $(2, 2)$ expresses numerous mixed modes of mimicry. It has two toxic, two non-toxic, and one predator types. The result will indicate how the additional equilibria affect the overall transitional dynamics. It will provide a glimpse of a more complex ecosystem. Results shed light on transitional pathways among equilibria.

(RL) Signal locking in system $(1, 0)$. We consider a population of two type groups: one set of 100 toxic type organisms, and one set of 100 predators. We initialize signal vectors for all toxic organisms by selecting a uniform random vector in $\Gamma = [-1, 1]^{15}$, and replicating this to all 100 members of the toxic type. We initialize the avoidance feature vector for the predators similarly, but hold the tolerance parameter throughout as $\tau = 0.2$. The initialization technique and geometry of higher dimensional space ensure with high probability that the signaling convention for avoidance must be learned, as opposed to being present initially. Notice also that the clonal strategies within type will be temporary as mutation will diversify the population with mutants. The mutation process selects each individual as a Bernoulli trial (with $p = 0.15$) for mutations. A strategy mutation occurs in place updating input vector $x \in \Gamma$ as $\tilde{x}$ distributed $\mathcal{N}(x, 0.1)$. The encounter process used in each time step is random pairing from the two types, thus each organism will encounter a randomly chosen predator.

In figure S5 we illustrate the evolutionary dynamics of signals and rewards. First, the distance between predator and prey vectors (in the encounters as a function of time) takes on a notable phase transition at around generation 800. The average cosine distance falls steeply and stays under the tolerance threshold value of τ, within the predator's avoidance region. At the same time, a transition in rewards sharply

increase. This establishes the signaling convention which satisfies both receiver and sender, and is the separating equilibrium of the signaling game. The equilibrium appears stable. Notice that both mutant predators and toxic organisms will probe various *out of equilibrium signaling strategies*; however, their lesser rewards will ensure their replication is outpaced by the better strategies in equilibrium. The differential in rewards is significant during replication, so once a separating equilibrium is established, the decreased rewards for mutants will act to strongly stabilize the separating equilibrium. Even though the equilibrium appears stable, its durability implicitly relies on mutation rate. Noting the extremes, when the mutation rates cool to zero the equilibrium will last indefinitely, whereas if there is too much mutation the equilibrium may not hold. Mutation rates have a sweet spot or region which is high enough to search for improvements and low enough to hold onto improvements once found.

Another insightful feature of this transition is that once the separating equilibrium is found, the cosine distance of interactions notably decreases in variance, this also reflects the stabilizing effect of the equilibrium's mutual increases in satisfaction for predator and prey alike, we find that whatever variance remains is primarily driven by mutation which vigilantly searches for possibly better equilibria. Below in figure S6 we illustrate how this signal locking occurs as a sequence of distinct activities.

Additionally measures on signal distance and path motion in the signal space are of interest because they indicate how signals evolve distinguishing *signal locking* from the stronger notion of *sample and hold.* In figure S7 we plot Intra and Extra variation with displacement to centroid measures. This allows us to evaluate how predator and toxic organisms adjust a population of signals. The greatest motion (measured by path distance of type centroids swept over time) is found prior to the transition to the separating equilibrium, there after decreases in residual variance and motion are observed. That motion still exists should indicate that the signal is somewhat fluid and moves according to co-evolutionary process.

(AD) Adversarial Dynamics in system $(0, 1)$**.** We consider a population of two type groups: one set of 100 non-toxic type organism, and one set of 100 predators. We initialize signal vectors for all toxic organisms by selecting a uniform random vector in $\Gamma = [-1, 1]^7$, and replicating this to all 100 members of the toxic type. We initialize the avoidance feature vector for the predators similarly, but hold the tolerance parameter throughout as $\tau = 0.2$. The initialization technique and geometry of higher dimensional space ensure that the non-toxic type will seek avoidance of its own protection, as opposed to being avoided initially. The clonal strategies within type will be temporary as mutation will diversify the population with mutants. The mutation process selects each individual as a Bernoulli trial (with $p = 0.15$) for mutations. A strategy mutation occurs in place updating input vector $x \in \Gamma$ as $\tilde{x}$ distributed $\mathcal{N}(x, 0.1)$. The encounter process used in each time step is random pairing from the two types, thus each organism will encounter a randomly chosen predator.

In figure S5 we illustrate the evolutionary dynamics of signals. Notice that the scenario is completely adversarial, whenever non-toxic type population approaches the predator avoidance region, the predator will evade the possibility of forming a separating equilibrium. The result illustrates the ease at which predator type can naturally evade and repel such hazards. In summary for equal size populations with identical mutation structures and a specific avoidance region it appears that Batesian type mimicry is no match for adaptive and reward oriented predators. Possible modulators which could change this outcome include: doubling of non-toxic mutation rates, doubling the non-toxic population size, halving the signaling space dimension,

doubling the predator's avoidance threshold, adjusting the metabolic reward.

(MR) Müllerian Ring emergent in system $(2, 0)$**.** We consider a population of two toxic types and one predator type, all composed of 100 organisms. For each type, we initialize signal vectors by selecting a uniform random vector in $\Gamma = [-1, 1]^7$, and replicate the selected vector to all 100 members of the type. The predator's tolerance parameter will be fixed throughout as $\tau = 0.2$. Similar to system $(1, 0)$, the initialization renders avoidance unlikely and needing to be learned due to high dimensional geometry. Also the inital clonality within types will be short lived due to mutation. The encounter process used in this experiment is like that of system $(1, 0)$, however we group the non predator types and compute six random pairs for each predator per time step for signaling game play. We use the co-mimic-A and co-mimic-B rewards for the types (see table 2).

Dynamics are shown in figure S9. Similar to system $(1, 0)$, Signal locking occurs between one of the two toxic types and predator first. While that equilibrium is reached first, the equilibrium is reinforced when the second toxic type joins the signal convention later to form textcolorredenhance the value of the signal a stronger separating equilibrium. Not surprisingly this suggests that the signal locking condition is an attractor, and maybe joined by multiple toxic types to further grow and reinforce the ring. It is still unclear what attributes of stability are improved by the addition of another type in the ring. For example, it appears from figure S10 that the motions of signal centroids is no slower with a mimicry ring than that of a single type.

(MM) Mixed mode mimicry yields an oscillator in system $(1, 1)$**.** We consider a population of one toxic type, one non toxic type and one predator type, all composed of 100 organisms. For each type, we initialize signal vectors by selecting a uniform random vector in $\Gamma = [-1, 1]^7$, and replicate the selected vector to all 100 members of the type. The predator's tolerance parameter will be fixed throughout as $\tau = 0.2$. Similar to system $(1, 0)$ and due to the high dimensional geometry, initialization renders the system in a state where predators need to learn toxic type, and non-toxic types have yet to exploit the predators avoidance. Also the initial clonality within types will be short lived due to mutation. The encounter process used in this experiment is like that of system $(2, 0)$, all non predator types are pooled for random pairing with predators, six random pairs for each predator per time step are generated for signaling game play. We use the model and mimic rewards for toxic and non-toxic type (see table 2).

The most notable characteristic of this system is oscillation. Below in Figure S11 we plot the dynamic characteristics of encounters and rewards. In S12 a more detailed view for a subset of time steps is given.

Our simulations suggest that there are two prominent cycles starting from babbling. The main progression appears to continue with partial pooling (mutual convention with toxic and predator types), pooling (when that convention is invaded by non-toxic type), and then back to babbling (when the Batesian mimics completely invert the predator benefits for maintaining the signal convention). The second progression occurs when the non-toxic type is the first to be avoided. This is followed by a rapid destabilization and return to babbling. We discuss these two progressions.

The main progression: The system is initialized to a babbling equilibrium, next a mutant toxic or predator type realizes a means of avoidance. Due to the boosted rewards the convention rapidly replicates in both the toxic and predator types to yield a partial pooling equilibrium as found in $(1, 0)$. This equilibrium is protected by natural selection, as the out of equilibrium signaling in either the predator or toxic

prey will result in less satisfying rewards and consequently be replicated at lower rates. Aside from low probability mutational events, the only disruptive factor is that non-toxic type could discover a mimetic strategy to exploit the avoidance for its own protection needs. Eventually the non-toxic type will invades causing transition to a pooling equilibrium. But that is short lived if the expected benefits of eating non-toxic prey outweighs the penalty for predation of toxic prey. In that case Mutations of the predator's avoidance feature are no longer deterred by selection, and a rapid relocation of the avoidance feature is expected. This competes the steps of the main progression the system returns to a babbling regime.

The second progression: Starting from babbling, the second progression starts with non-toxic type being first to be avoided, this can occur when the non-toxic type mutates an individual that exploits the avoidance features of predator. When this happens, predators quickly responds with its own mutants that adjust avoidance to expel the non-toxic type from its avoidance region, thus returning to babbling in order to reestablish the predation benefit. The dynamics are essentially that of system $(0, 1)$ and are independent of the behavior of toxic type that is occupied with search.

(AD2) Adversarial learning and aversion in system $(0, 2)$. We consider a population of two non-toxic types and one predator type, all composed of 100 organisms. For each type, we initialize signal vectors by selecting a uniform random vector in $\Gamma = [-1, 1]^7$, and replicate the selected vector to all 100 members of the type. The predator's tolerance parameter will be fixed throughout as $\tau = 0.2$. The encounter process will group the non predator types and compute six random pairs for each predator per time step for signaling game play. We use the mimic rewards for the both non-toxic types (see table 2).

Dynamics are shown in figure S13. Note that the dynamics are adversarial. In system $(0, 1)$, the predator, reserving its avoidance region to mark toxic organisms, protects against nutritious non-toxic prey entering. System $(0, 2)$ is similar but is two such independent components played at once against the predator. We conclude that if the signal space is reasonably large the predator will be able to avoid a large number of such types.

(MRA) Stability of the Müllerian Ring to Batesian invasion in system $(2, 1)$. We consider a population of two toxic, one non-toxic and one predator type, all composed of 100 organisms. For each type, we initialize signal vectors by selecting a uniform random vector in $\Gamma = [-1, 1]^7$, and replicate the selected vector to all 100 members of the type. Additionally the predator's tolerance parameter will be fixed throughout as $\tau = 0.2$. The encounter process used in this experiment will group the non predator types and compute six random pairs for each predator per time step for signaling game play. We use the mimic, co-mimic-A and co-mimic-B rewards for the types (see table 2).

In figure S14 we illustrate the evolutionary dynamics of signals and rewards. Similar to the behavior of $(2, 0)$ we observe the formation of a ring. The ring however lacks resiliency to withstand the invasion of a Batesian Mimic. Interestingly we observe an extensive period of uncertainty (during time steps 850 to 1600). The system appears not fully in pooling nor able to eject the Batesian invader from the predator's avoidance region. Eventually the system restarts to a babbling equilibria. Additionally (after time step 2400) we observe that while toxic type 1 has drifted far from avoidance, toxic type 2, non-toxic type and predator seem to act very similar as they might in system $(1, 1)$.

(S12) Mostly adversarial dynamics in system $(1, 2)$ with slight reprieve of short lived signal locking with toxic type. . We consider a population of one toxic, two non-toxic and one predator type, all composed of 100 organisms. For each type, we initialize signal vectors by selecting a uniform random vector in $\Gamma = [-1, 1]^7$, and replicate the selected vector to all 100 members of the type. Additionally the predator's tolerance parameter will be fixed throughout as $\tau = 0.2$. The encounter process used in this experiment will group the non predator types and compute six random pairs for each predator per time step for signaling game play. We use the mimic, co-mimic-A and co-mimic-B rewards for the types (see table 2).

Dynamics are shown in figure S15.

When we compare the dynamics of this system with that of $(2, 1)$ we notice substantially less time spent in any signal locking state, and this policy of blanket avoidance seems to anticipate the predator behavior.

(MM2) Complex equilibria transitions in system $(2, 2)$. We consider a population of two toxic, two non-toxic and one predator type, all composed of 100 organisms. For each type, we initialize signal vectors by selecting a uniform random vector in $\Gamma = [-1, 1]^7$, and replicate the selected vector to all 100 members of the type. Additionally the predator's tolerance parameter will be fixed throughout as $\tau = 0.2$. The encounter process used in this experiment will group the non predator types and compute six random pairs for each predator per time step for signaling game play. We use the mimic, co-mimic-A and co-mimic-B rewards for the types (see table 2).

We illustrate the dynamics are shown in figure S16.

We note that with system $(2, 2)$ we see many of the dynamics from constituent systems, formation of the Ring, destabilization by a mimic, reformation and multiple invasions. Despite the complexity of the dynamics the transitions of equilibria can be understood in terms of our basic image presented 5. Rather than having two partial pooling equilibria (one for locking signals with toxic type, the other for locking signal with non-toxic type) We may view the separating equilibria with a binary relation on four types. Separating equilibria transitions can be done by change of relation in one of the types. The babbling equilibria will correspond to absence of signal locking relation for all the types. This then cases the pooling equilbria as separating equilibria that contain a relation with at least one toxic and at least one non-toxic type. The strongest Müllerian ring is composed of a separating equilibria with all members of the toxic type and without any members of the non-toxic class.

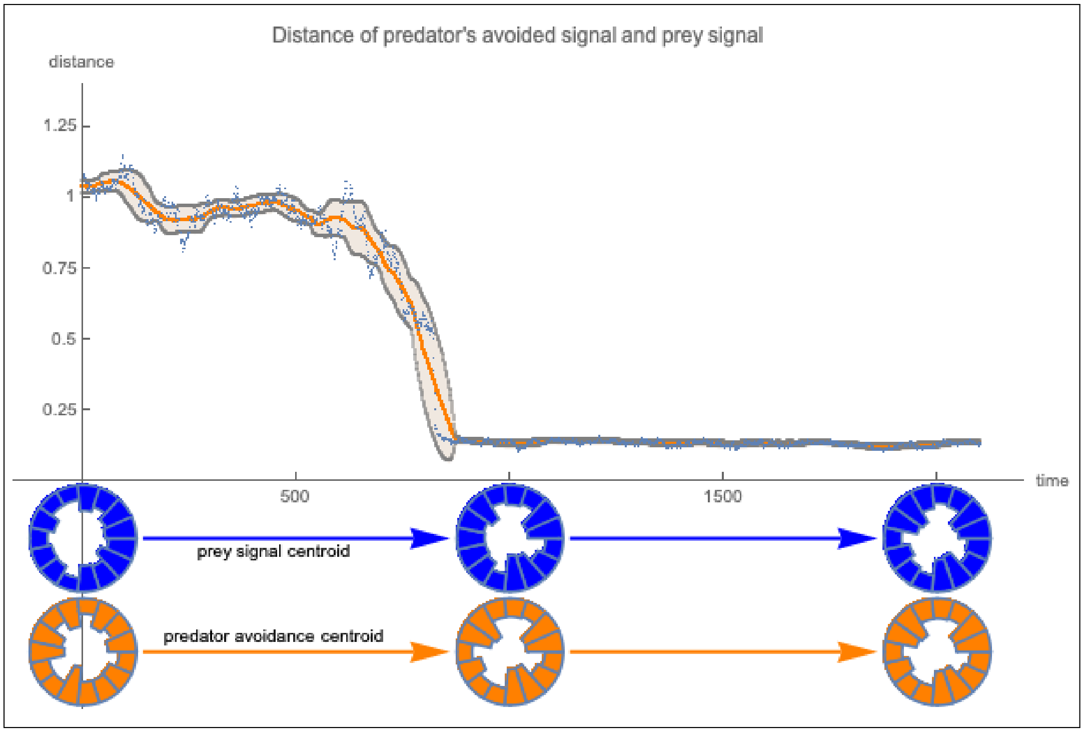

(a)

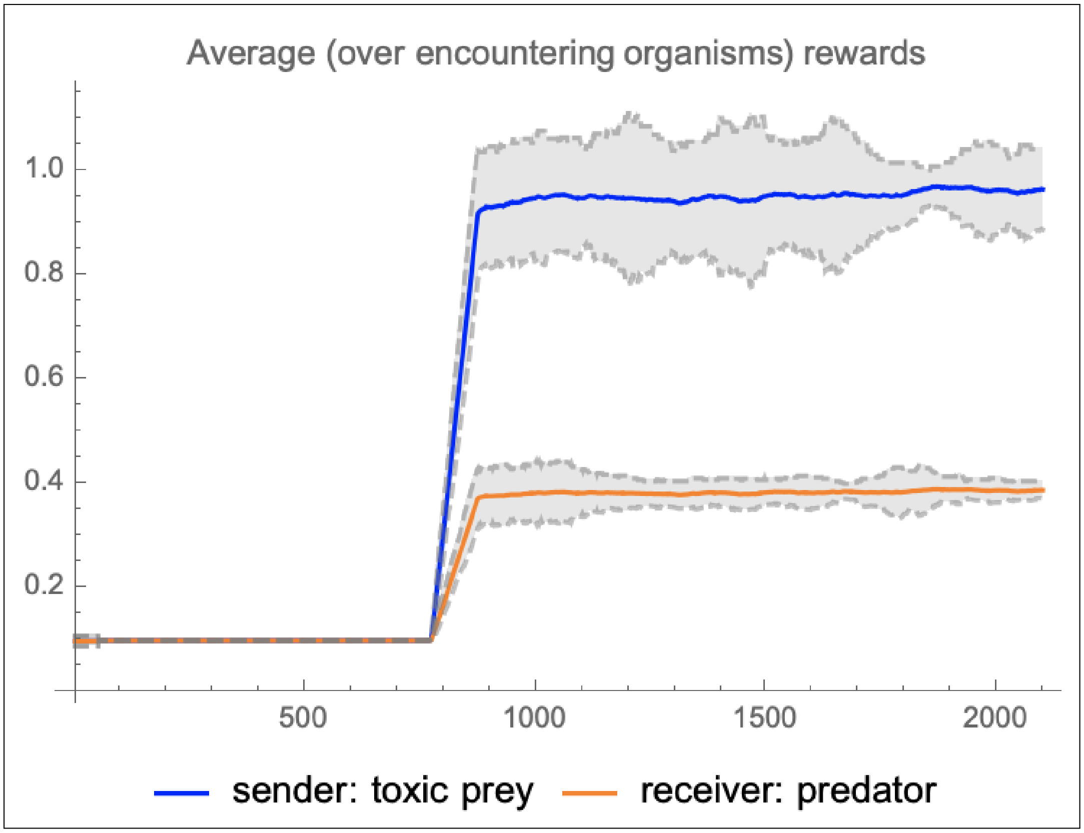

(b)

Fig. S5: A Signaling system with separating equilibrium evolves following a phase transition in both cos distance of encounters and bulk utility per species. We further observe that the separating equilibrium appears dynamically stable in time. The signal space is: $\Gamma = [-1, 1]^{15}$

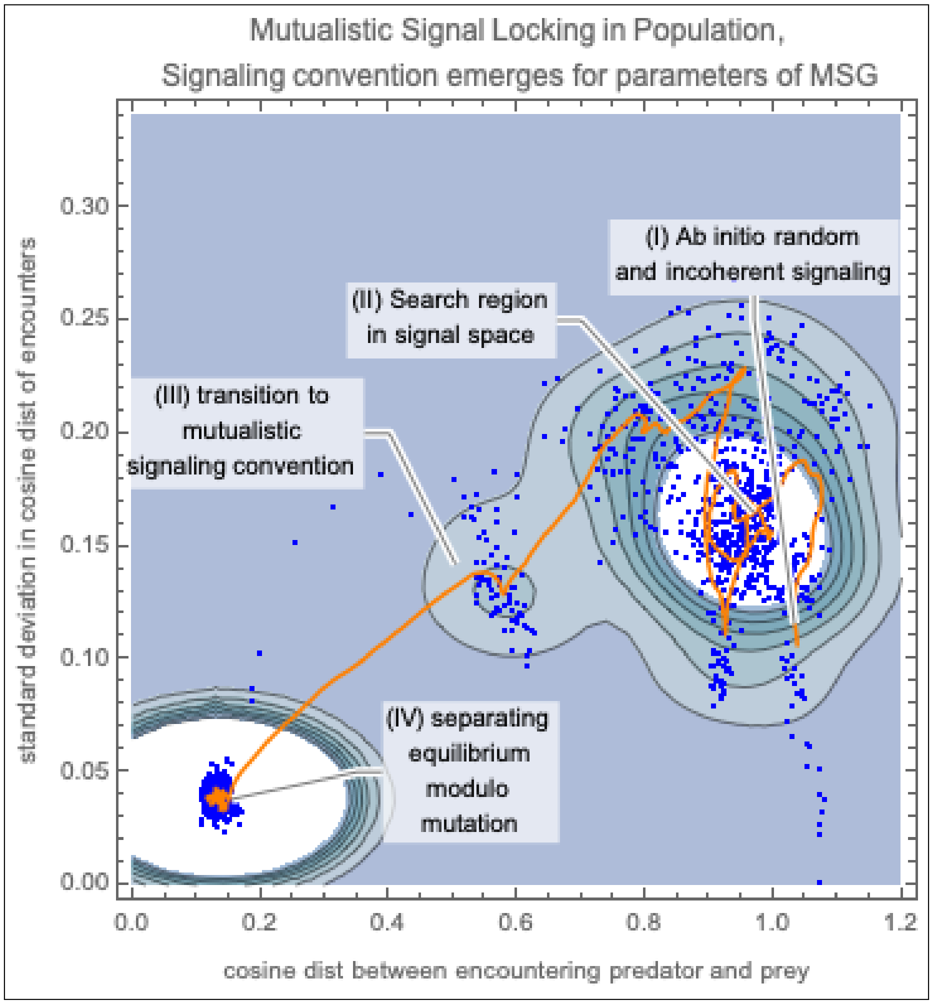

Fig. S6: Evolutionary games results in separating equilibrium in the common interest to both predator and toxic organism. The evolutionary game history begins with babbling or incoherent signaling, mutation drives search for both types until at least one predator and toxic type organism realize a meaningful signaling/avoidance convention, this leads to a transition in population strategies to yield a steady and stable separating equilibrium. In this example the the signal space is: $\Gamma = [-1, 1]^7$.

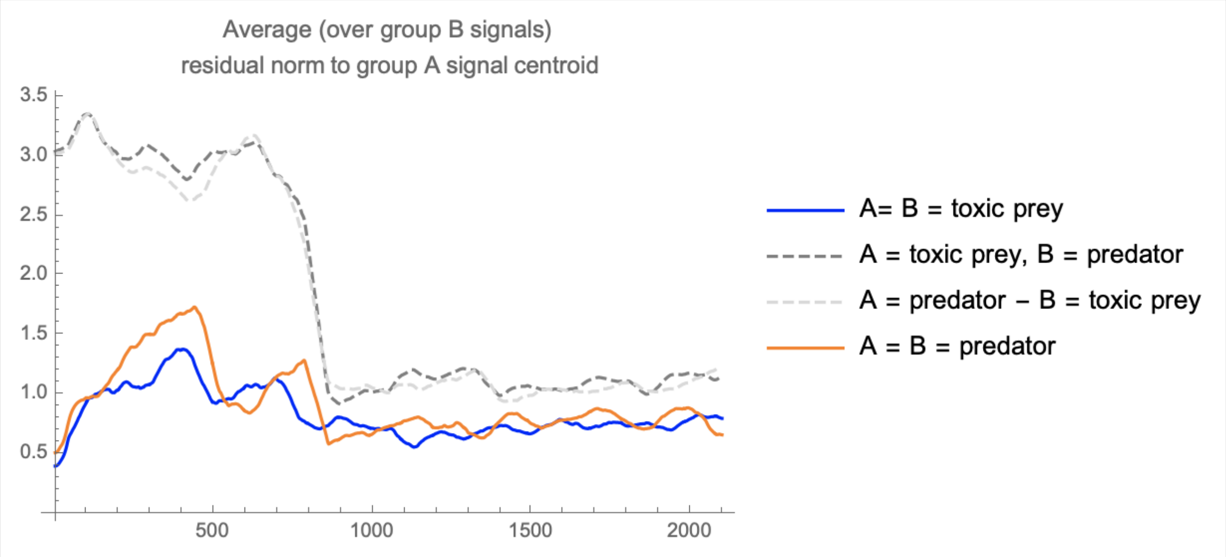

(a)

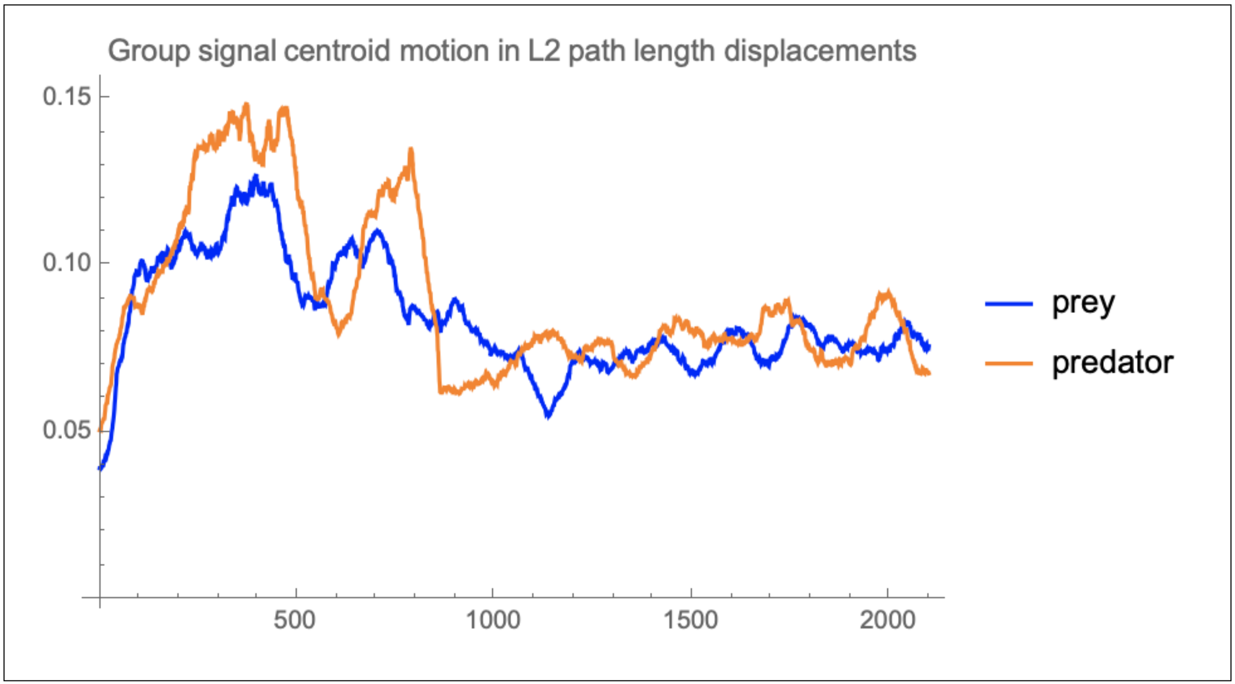

(b)

Fig. S7: System $(0, 1)$ learns. Variation and motion in Signal Space: Above in (a) the intra-type variations as a function of time are plotted for toxic type (blue) and predator (orange), Additionally the extra-type variations illustrate clearly the transition to an honest signaling purpose (i.e., separating equilibrium). The centroid path variation for each type signaling is plotted below in (b). Its worth noting that both toxic and predator types appear to have similar characteristics, suggesting that the signaling convention meets in the middle. Throughout this learning is done in $\Gamma = [-1, 1]^{15}$.

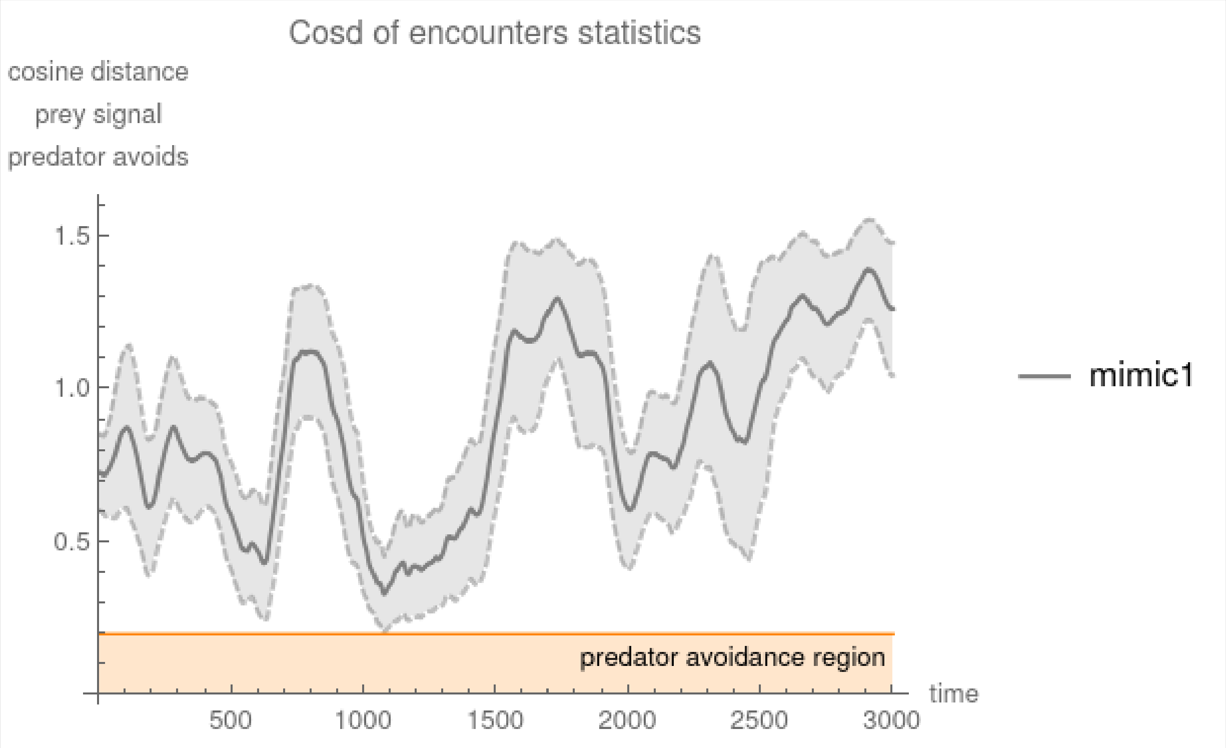

(a)

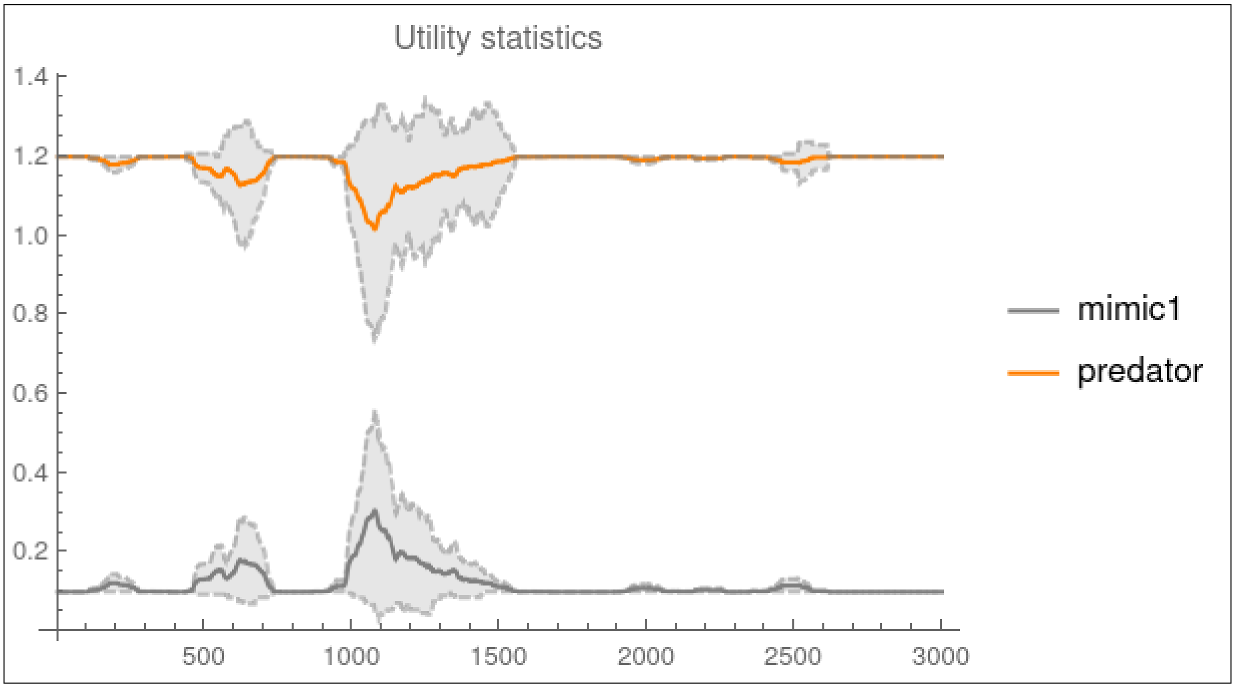

(b)

Fig. S8: Aversion dynamics. The predator and the non-toxic types have opposing goals, predator seeks its metabolic requirements at the expense of the non-toxic type, while non-toxic type organisms seek protection at the expense of the predator. There is no common goal so the dynamics are adversarial. The signaling convention is repelled by the predator, when the non-toxic type learns and exploits the predator's avoidance region the predator immediately updates obscures region. Throughout $\Gamma = [-1, 1]^7$.

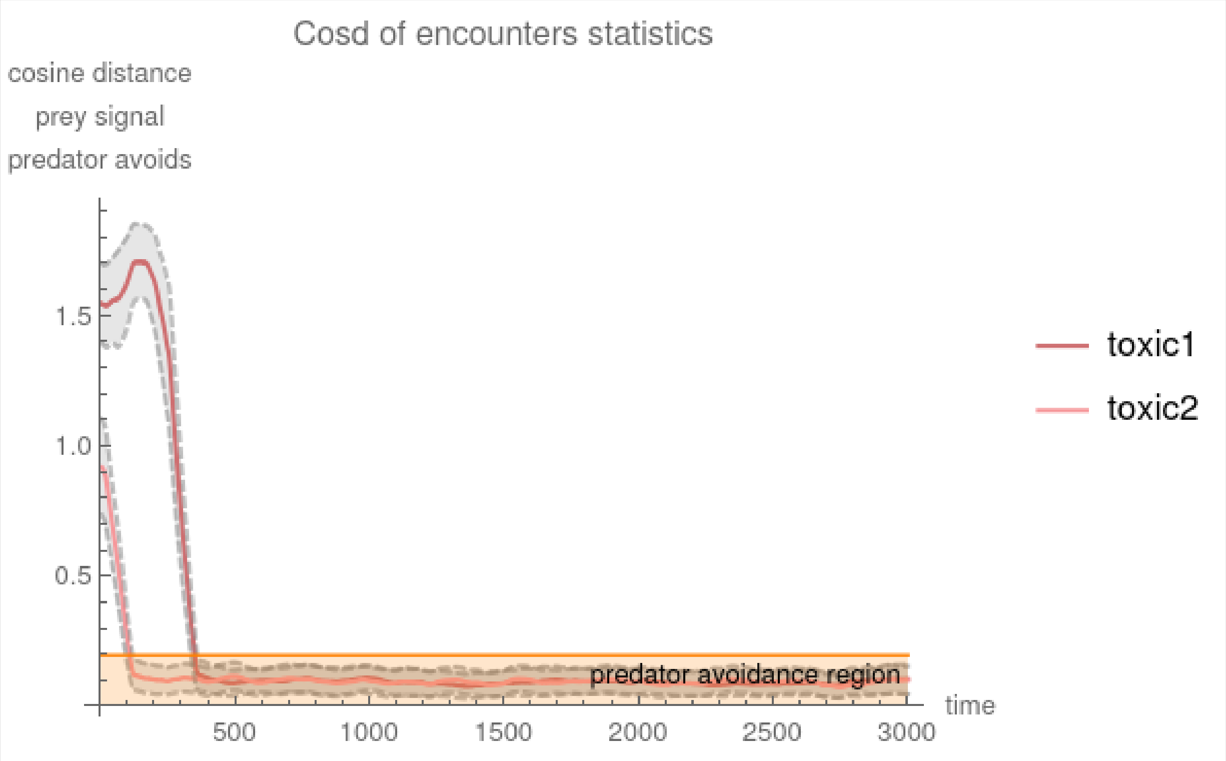

(a)

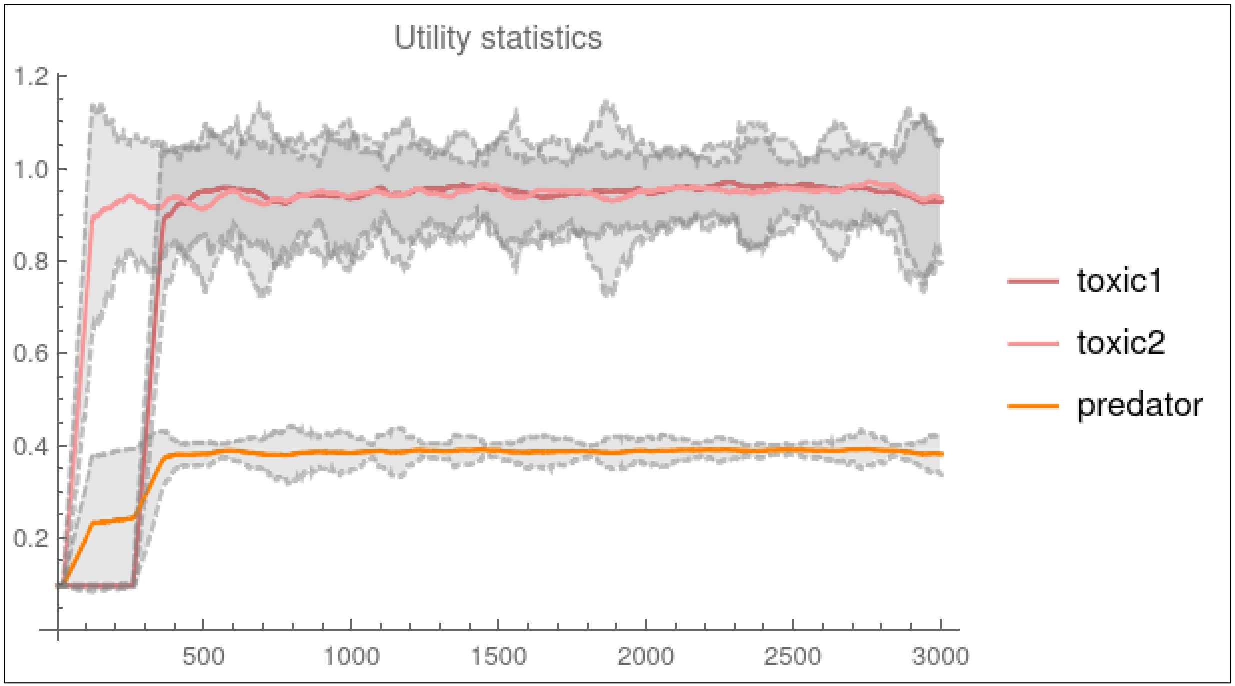

(b)

Fig. S9: Formation of a Müllerian ring. The signaling system first finds a separating equilibrium between toxic type 2 and predator, formed around generation 250. The equilibrium transitions to a partial pooling equilibrium reinforced by toxic type 1 around generation 650, when the ring is formed. The equilibrium appears to be dynamically stable. Throughout the signal space is: $\Gamma = [-1, 1]^7$

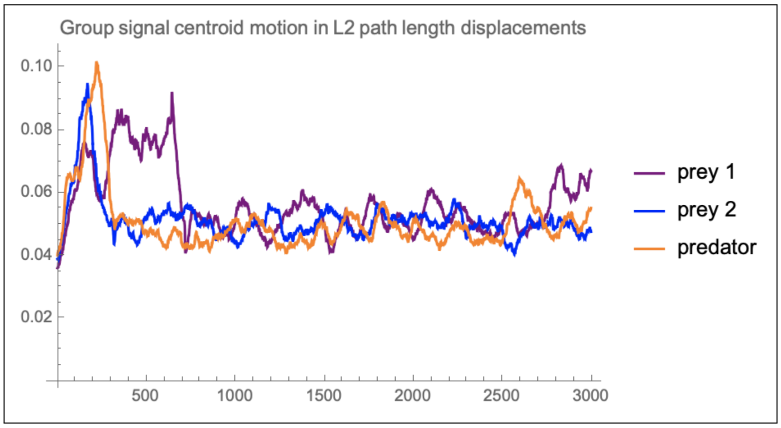

(a)

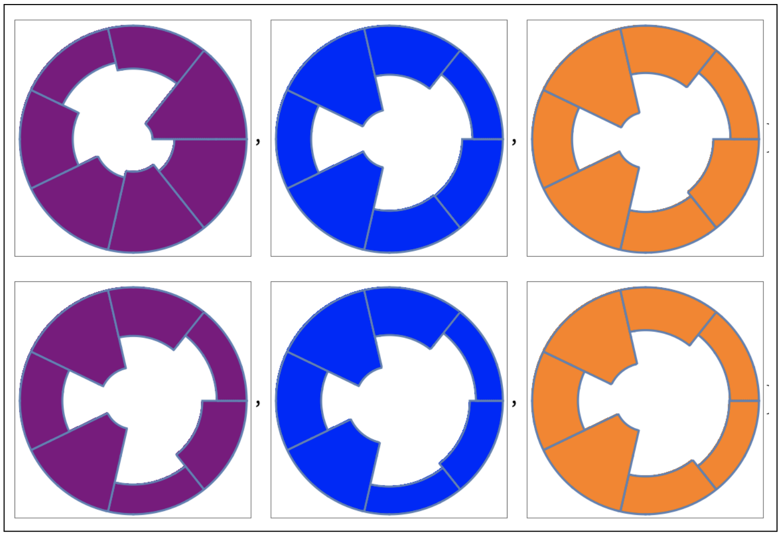

(b)

Fig. S10: Signal centroid motions as species search for separating equilibrium. Notice that the the motion for predator and prey-2 slows after a shared equilibrium is found around generation 250. On the other hand the motion of prey-1 remains high until it joins the equilibrium signal. Samples of signals are viewed in (b) where the top row is the mean signals for (prey1, prey2, predator) at generation 500. Notice that prey 2 and predator are similar but prey 1 is not. In the bottom row the mean signals are shown again for generation 1300, notice that prey 1 (purple) transitions and all signals look alike. Throughout the signal space is: $\Gamma = [-1, 1]^7$.

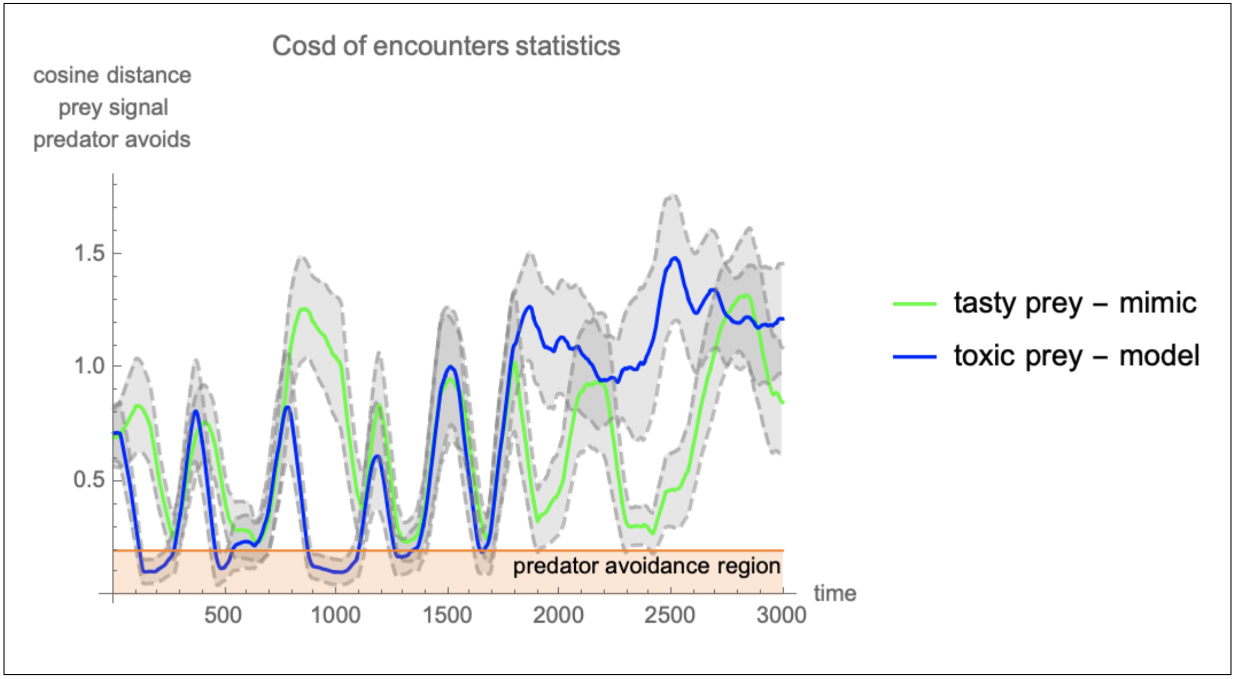

(a)

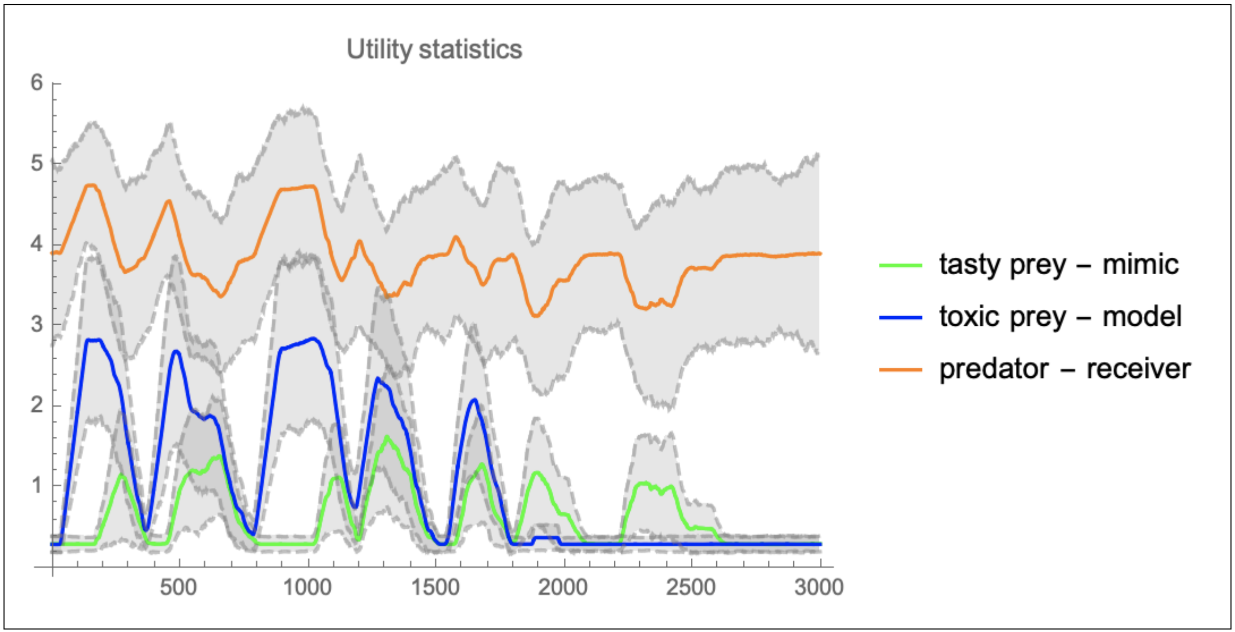

(b)

Fig. S11: A Signaling system Oscillator. In a system with one toxic, one non-toxic and one predatory type an oscillation is observed. The system is initialized from a babbling equilibrium. The sequence continues in search, followed by toxic type securing a separating equilibrium. From this equilibrium, the nourishing non-toxic type invades as Batesian mimic, this partial pooling equilibrium doesn't appear to be dynamically stable as these invasions seem to ejects the convention in favor of a babbling equilibrium.

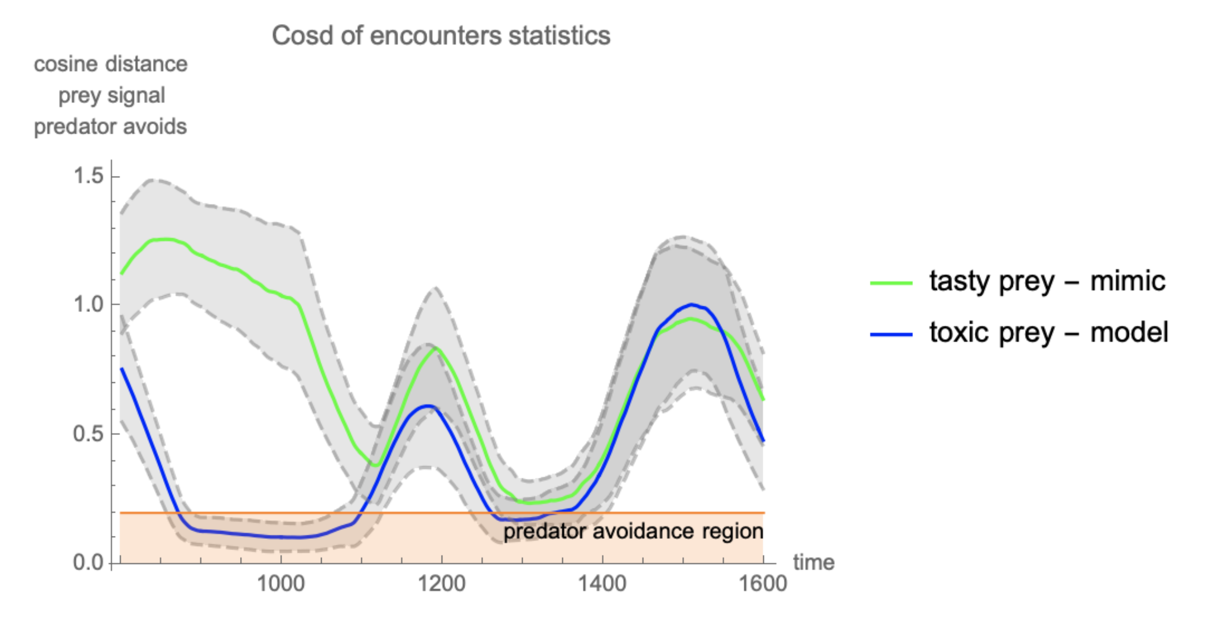

(a)

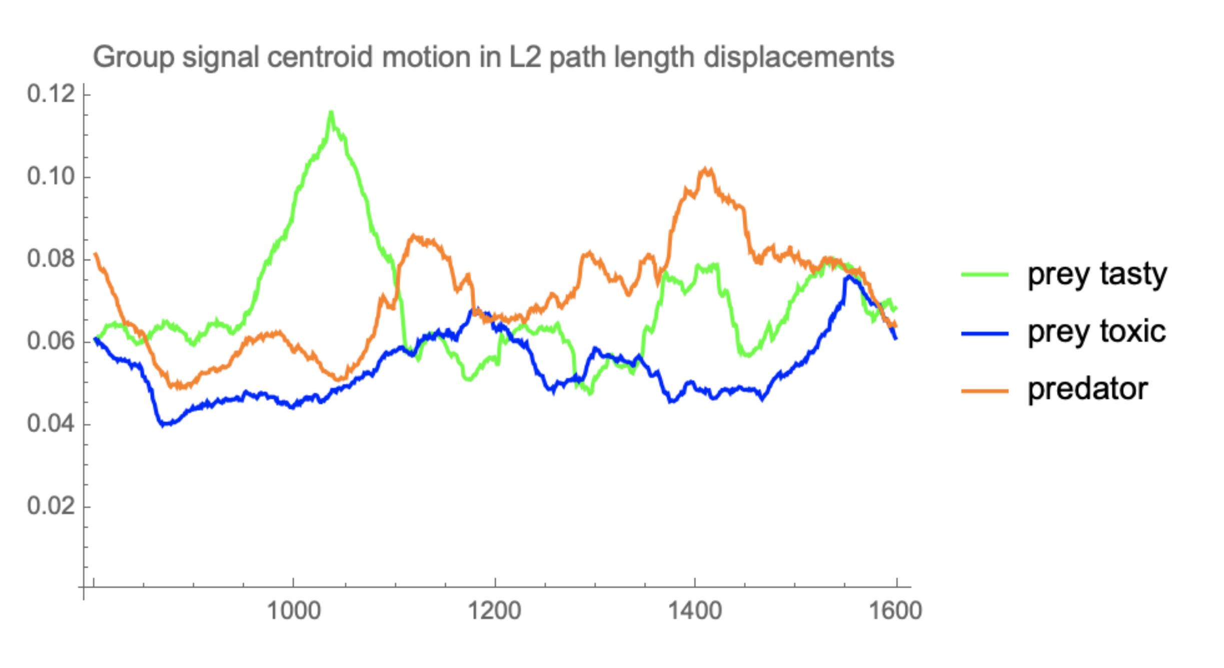

(b)

Fig. S12: A detailed view of the Memetic Oscillator. In (a) the main progression can be seen, but also of interest are the cooresponding centroid path movements in (b). We observe that Batesian invasion seems to precipitates an increase in preator path movement, thus suggesting urgency of return to babbling.

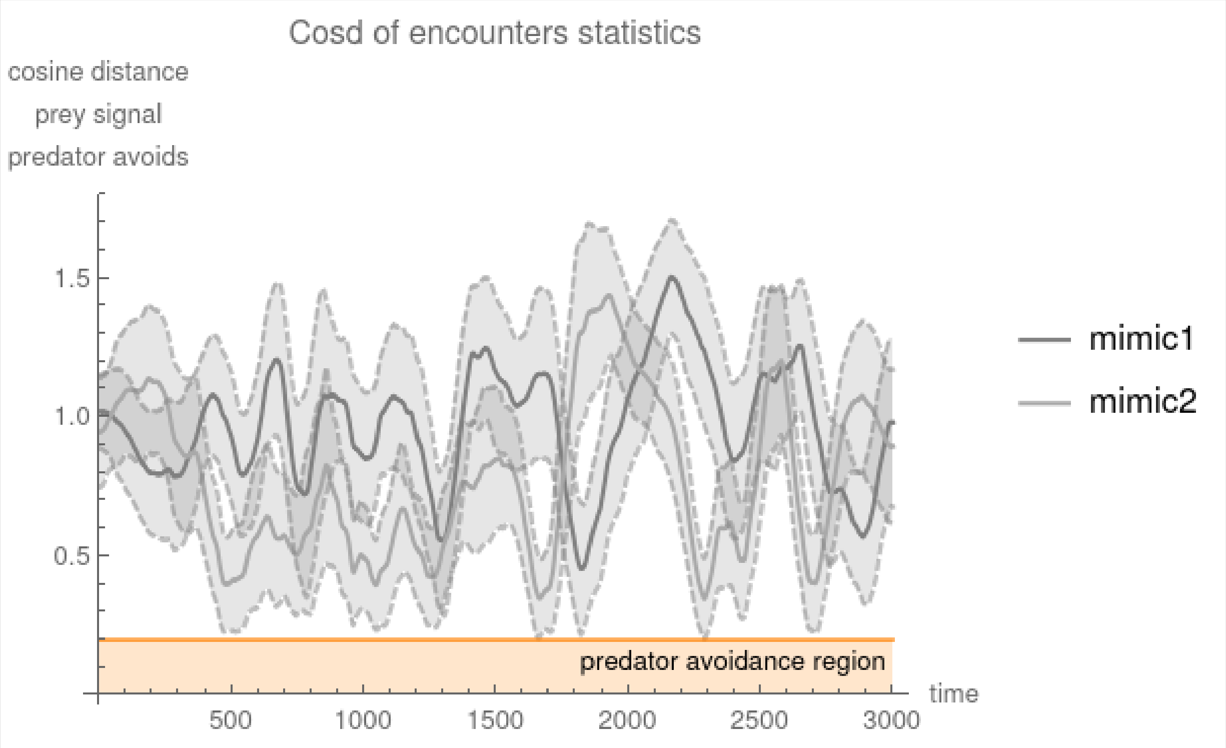

(a)

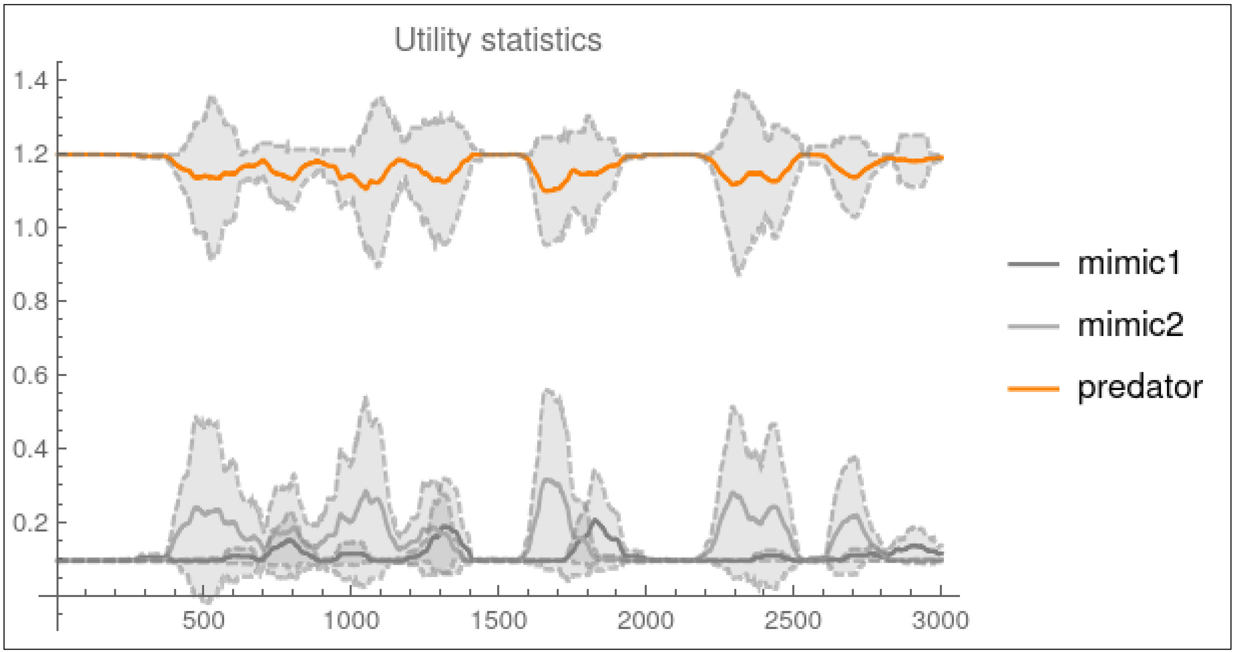

(b)

Fig. S13: Repulsion to invasions. In system $(0,2)$ predators must stabilize their rewards by preventing non-toxic types entry to an avoidance region, reserved for toxic types. The dynamics are adversarial, where non-toxic mimics probe for a predator's vulnerability, and predator adjusting defenses to prevent being exploited. The constant probe and evasion is illustrated by the cosine distance plotted in (a). The corresponding reward functions which drive replication are plotted over the same time in (b). Throughout the signal space is: $\Gamma = [-1,1]^7$

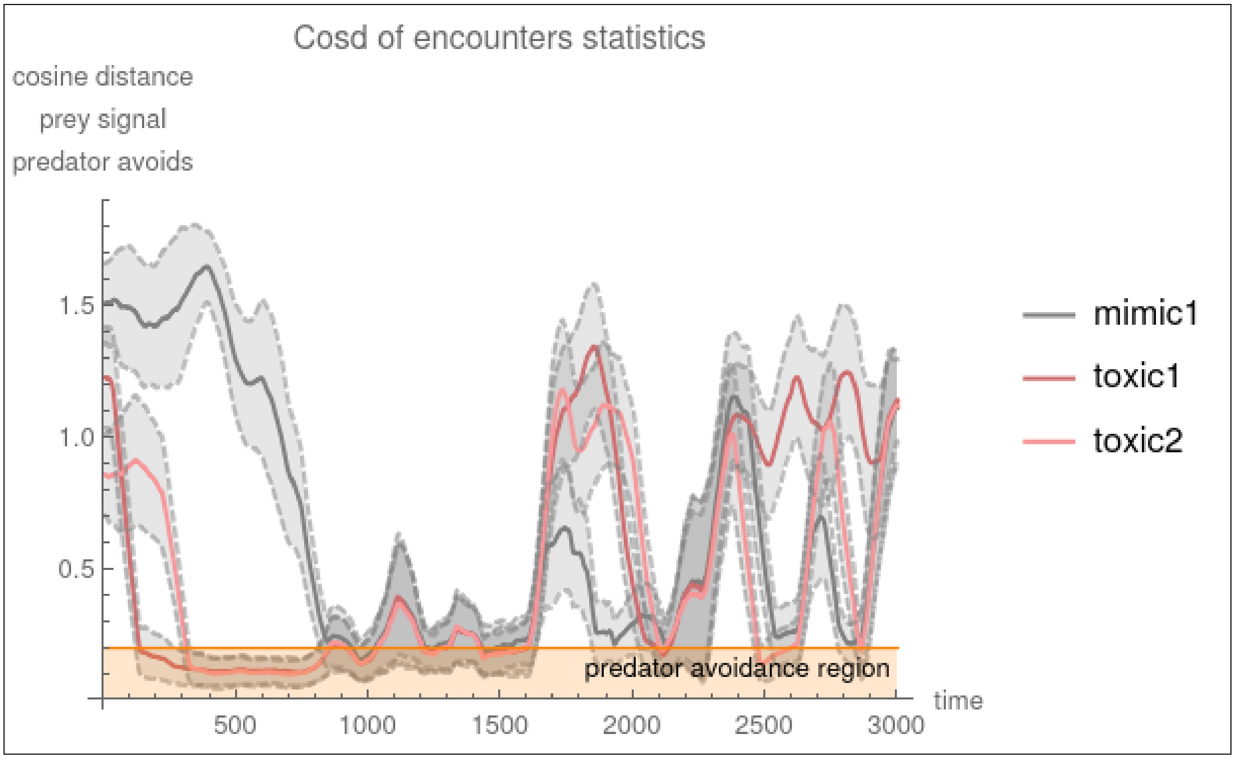

(a)

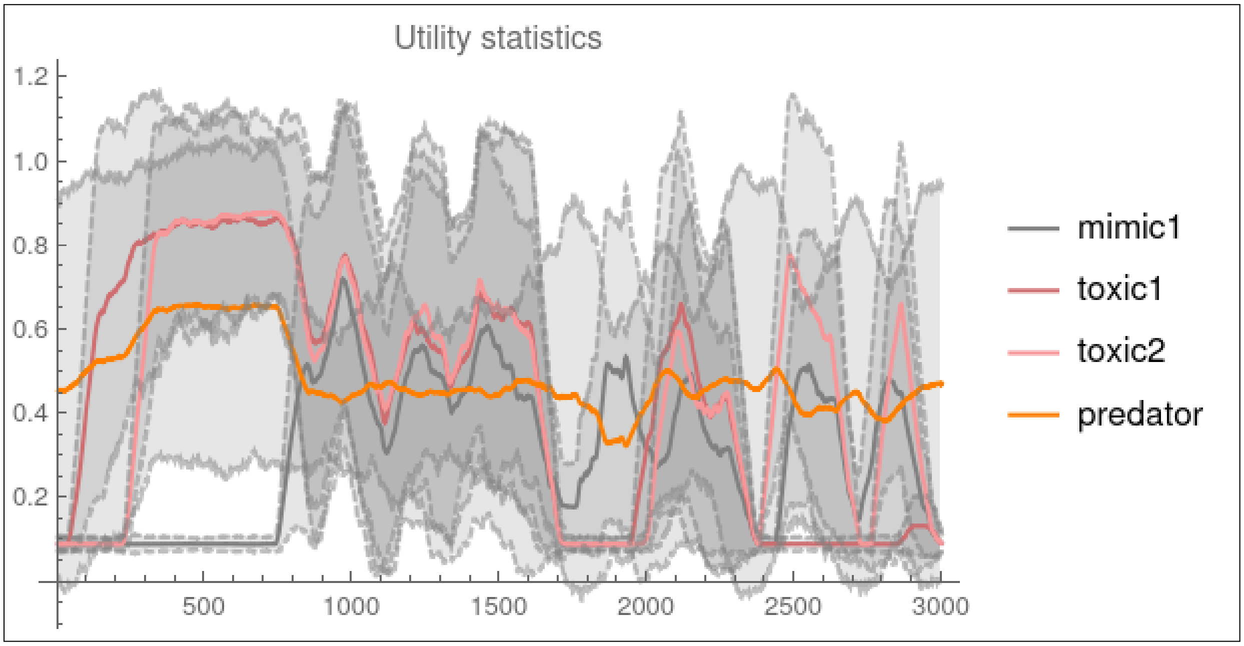

(b)

Fig. S14: Stability of a Müllerian ring will depend on the Batesian invasion. The signaling system (a) first finds a separating equilibrium with toxic type 1 and predator (formed around time step 100). Next the toxic type 2 joins to create a partial pooling ring (around time step 330), The ring is stable until (near time step 800) it is invaded by non-toxic type. The ring appears destabilized but neither broken nor restored fully, until finally reaching a full babbling equilibrium (near time step 1700). The corresponding reward functions over the same time are plotted in (b). Throughout the signal space is: $\Gamma = [-1, 1]^7$

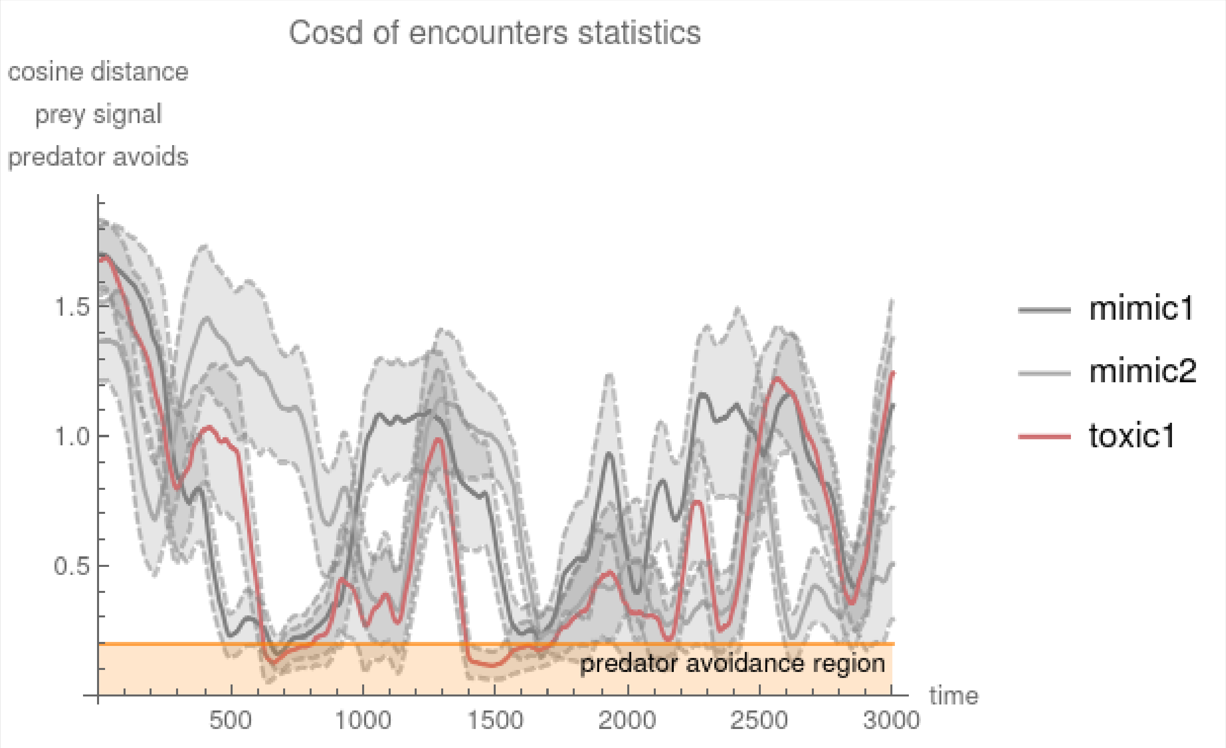

(a)

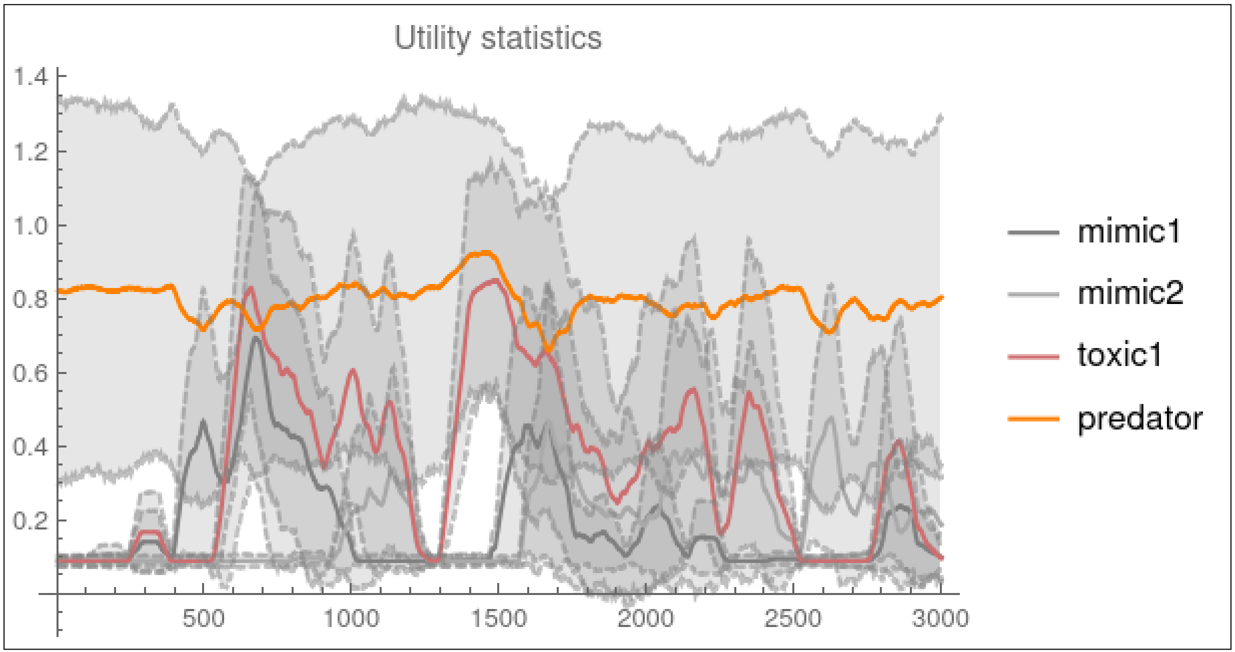

(b)

Fig. S15: System (1, 2) predators must stabilize their rewards by preventing and one or both of the non-toxic types entry to an avoidance region, reserved for the toxic types. The dynamics are adversarial, with slight moments of reprieve when predator can lock to toxic organisms signal. However these signal locking epochs are not long lived, due to invasion from non-toxic types. The cosine distance of encounters are plotted in (a). The corresponding reward functions which drive replication are plotted over the same time in (b). Throughout the signal space is: $\Gamma = [-1, 1]^7$

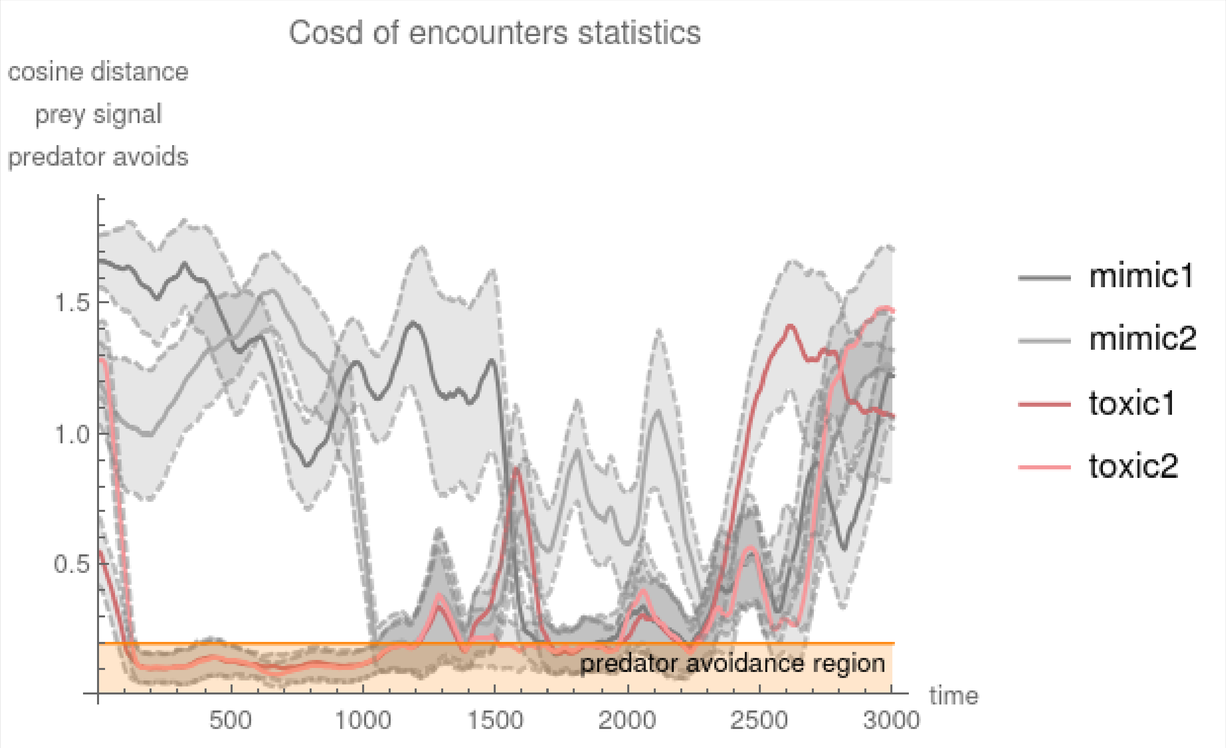

(a)

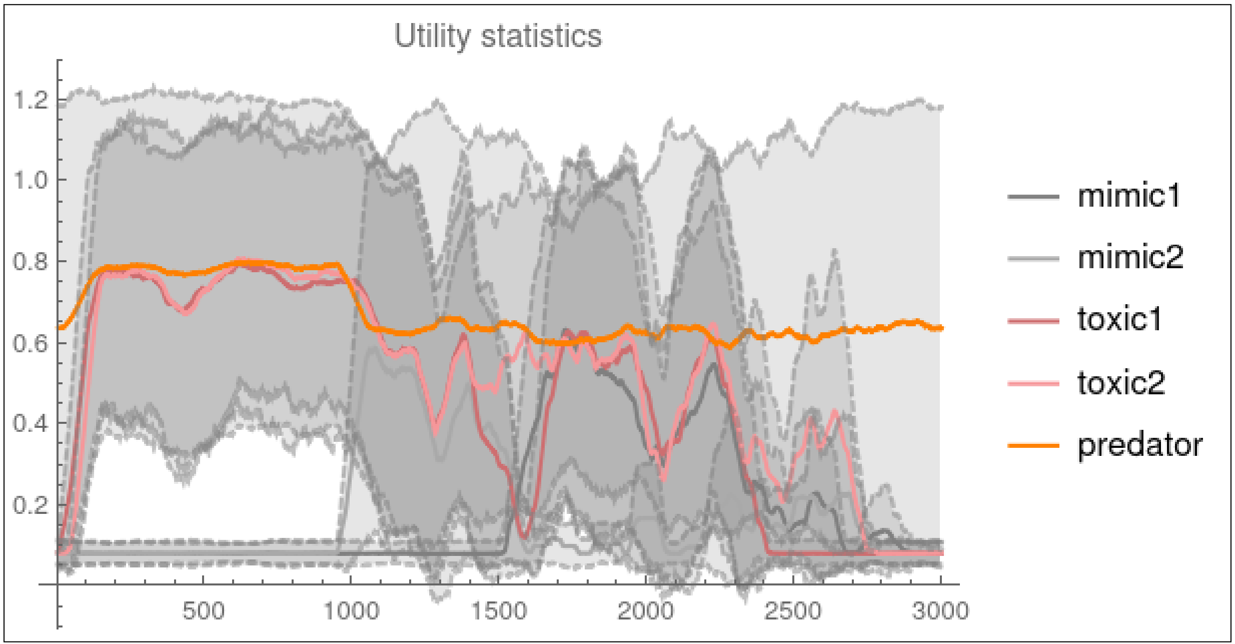

(b)

Fig. S16: System $(2, 2)$ offers a variety of equilibria, predator prevent one or both of the non-toxic types entry to an avoidance region, reserved for one or both toxic types. The dynamics are complex sampling from constituent systems. The cosine distance of encounters are plotted in (a). The corresponding reward functions which drive replication are plotted over the same time in (b). Throughout the signal space is: $\Gamma = [-1, 1]^7$
